# Life-cycle plasticity enables conditional asexual reproduction in a kelp

**DOI:** 10.1371/journal.pbio.3004065

**Published:** 2026-11-12

**Authors:** Daniel Liesner, Rémy Luthringer, Lineekela Kandjengo, John J. Bolton, Mark D. Rothman, Myriam Valero, Fabian B. Haas, Susana M. Coelho

**Affiliations:** 1 Algal Development and Evolution, Max Planck Institute for Biology Tübingen, Tübingen, Germany; 2 Department of Fisheries and Ocean Sciences, University of Namibia, Henties Bay, Namibia; 3 Department of Biological Sciences, University of Cape Town, Rondebosch, South Africa; 4 Department of Forestry, Fisheries and the Environment, Vlaeberg, Cape Town, South Africa; 5 UMR 7144, CNRS, Sorbonne Université, Dispersal Speciation and Evolution of Marine species, Station Biologique de Roscoff, Roscoff, France; Duke University Medical Center, UNITED STATES OF AMERICA

## Abstract

Many organisms alternate between distinct life stages and can reproduce either sexually or asexually, yet the mechanisms coordinating these developmental and reproductive transitions remain poorly understood. Here, we investigate the molecular basis of life stage identity and reproductive mode in the kelp *Laminaria pallida*, a multicellular organism with a complex life cycle. By combining gene expression analyses with developmental experiments and natural population-level approaches, we characterize the transcriptional signatures associated with developmental state transitions in both sexual and asexual contexts. We show that asexual reproduction is associated with endoreduplication and reduced fitness, while it is retained but rarely expressed in two natural populations. Moreover, we find that the presence of potential mating partners reduces asexual development, revealing that reproductive mode is adjusted in response to reproductive opportunities. Together, these findings suggest that asexual reproduction functions as a conditional alternative to sex, promoting persistence when sexual reproduction is constrained while preserving the long-term benefits of sexual reproduction when mates are available. More broadly, our results reveal how organisms integrate developmental, environmental, and reproductive cues to balance alternative reproductive strategies across complex life cycles.

## Abstract 2

Many organisms can reproduce sexually or asexually, but how reproductive mode is coordinated across complex life cycles remains unclear. This study shows that kelp adjust reproduction to mating opportunities, with asexuality serving as a conditional persistence strategy when sex is constrained.

## Introduction

Sexual reproduction is widespread among eukaryotes and plays a central role in evolutionary processes by reshuffling parental genomes and generating new combinations of traits upon which natural selection can act [1]. In comparison with asexual reproduction, however, sexual reproduction entails considerable costs relating to, e.g., meiosis [2] or the production of males [3]. Sexuality has indeed independently been lost across many eukaryotic lineages [4,5]. While obligate asexuality is rare in animals and plants, other lineages such as fungi or many unicellular eukaryotes have never been observed to reproduce sexually [4]. The respective alternative life cycle strategies bypass meiosis and/or syngamy in processes such as parthenogenesis, apomixis, fragmentation or vegetative propagation [6,7]. Shifts between sexuality and asexuality may also occur within a single species and are often associated with life history trade-offs, developmental plasticity, or ecological adaptation [8,9], e.g., in geographical parthenogenesis at distributional range edges [10]. Facultative asexuals, which are able to switch between reproductive modes in a plastic response, offer a powerful system to explore the genetic and environmental controls governing reproductive mode transitions.

Brown algae (Phaeophyceae) provide a powerful system for studying reproductive mode switching because most species have complex life cycles and can reproduce both sexually and asexually [11–14]. In the brown alga *Scytosiphon*, female gametes develop parthenogenetically in natural populations, underscoring the ecological significance of asexual reproduction in this lineage [15]. This process is controlled by both the U sex chromosome and an autosomal locus [15]. A role for sex chromosomes in parthenogenesis has also been demonstrated in the model brown alga *Ectocarpus* [11]. In *Ectocarpus*, the sporophyte developmental program is activated in both zygotes and unfused gametes through the dimerization of two TALE homeodomain transcription factors [16,17], uncoupling life-cycle identity from ploidy. As a result, both female and male gametes can develop parthenogenetically into haploid parthenosporophytes. Although these individuals acquire sporophyte identity, previous observations suggest that parthenosporophyte development does not fully recapitulate that of diploid, zygote-derived sporophytes [11,18]. However, the extent to which the developmental and transcriptional programs initiated during parthenogenesis resemble those of sexual embryogenesis, and where they diverge, has not been systematically investigated.

Morphologically complex brown algae such as kelps also exhibit pronounced plasticity in their life cycles and reproductive modes. As ecologically important marine foundation species in diverse and highly productive coastal ecosystems [19], kelps display a marked contrast between their microscopic, filamentous gametophyte stage and their large, structurally complex sporophytes, which can reach several meters in length. Gametes are strongly dimorphic: small, motile sperm released by male gametophytes fertilize large, immotile eggs produced by females, initiating zygotic embryogenesis (Fig 1A). Notably, unfertilized female gametes (eggs) can undergo parthenogenetic development [20–22], although these parthenosporophytes are often morphologically abnormal and exhibit reduced fitness compared to sexually derived sporophytes [23–25]. Despite the potential ecological and evolutionary relevance, the molecular mechanisms governing reproductive mode transitions and plasticity in kelps, and particularly the regulation of gametophyte-to-sporophyte developmental switches remain largely unknown.

**Fig 1:**
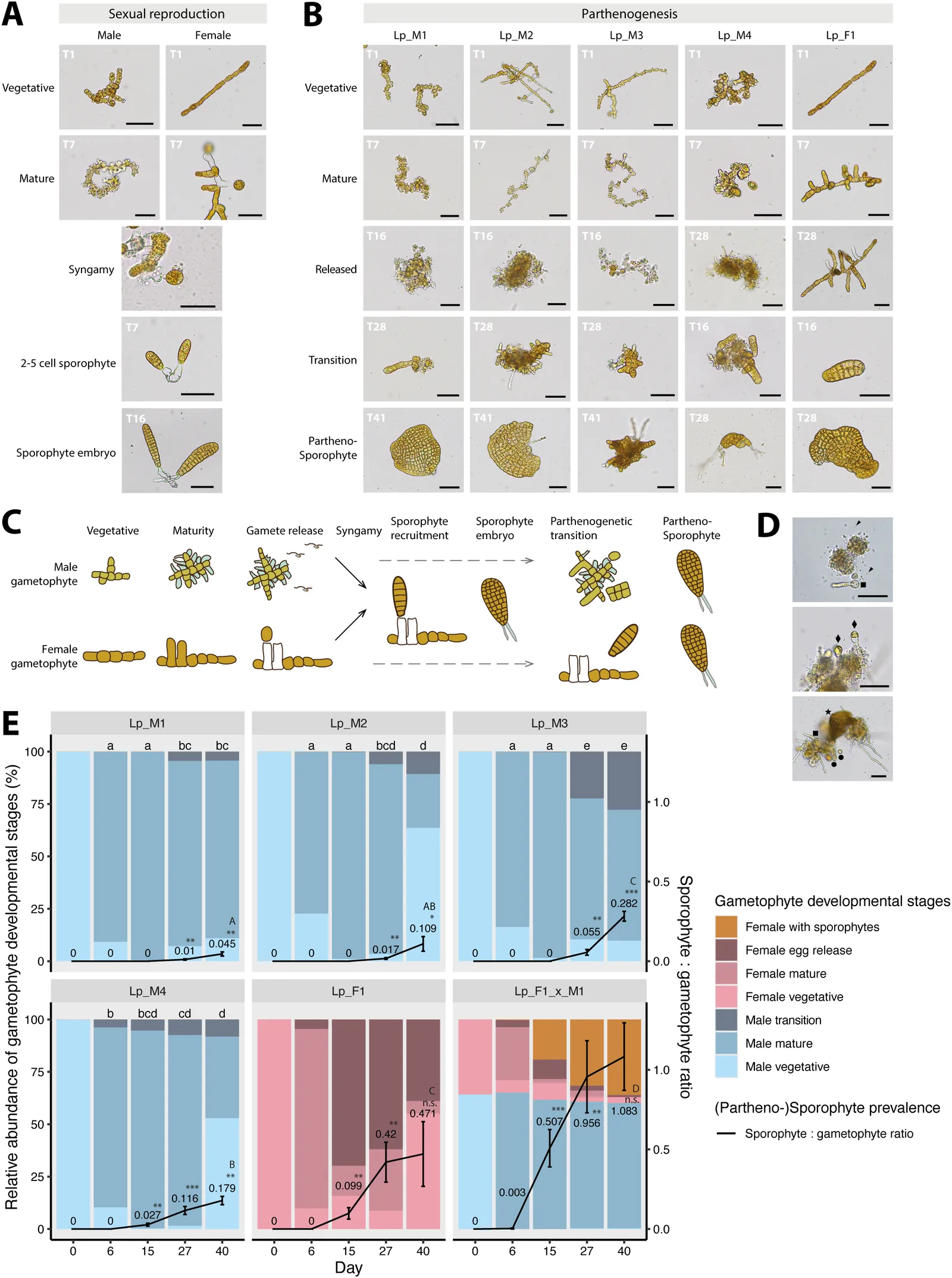
Developmental time course of *Laminaria pallida* gametogenesis and (partheno-)sporophyte production. **(A)** Three developmental stages were analysed during sexual reproduction of male and female gametophytes (vegetative, mature, female egg) and two stages of sporophyte embryo development (2-5 cell sporophyte, sporophyte embryo). **(B)** Five developmental stages during parthenogenetic development were analysed (vegetative, mature, after gamete release, morphological transition / early parthenosporophyte tissue development, parthenosporophyte) and collected at five time points (T1, T7, T16, T28, T41). Parthenogenesis occurred in all four male gametophytes (Lp_M1 – Lp_M4) and in the female Lp_F1. Note that gametophytes of Lp_M1 which did not transition were used as a control for all time points. Additionally, sporophytes developed from sexual reproduction of Lp_F1 and Lp_M1 were used as comparisons. **(C)** Schematic depiction of sexual and parthenogenetic reproduction in *L. pallida*, partially modified from [30] published under CC BY 4.0. **(D)** In addition to sperm (arrowheads) male gametophytes produce enlarged gametangia (diamonds) which release egg-like gametes (circles) which “germinate” (squares) and grow into parthenosporophytes (star). **(E)** Relative abundance of parthenosporophytes in cultures of four male strains as compared to a female line and to the presence of sexually derived sporophytes. Average relative abundance of developmental stages over time is shown in stacked bar plots (n = 5 replicate dishes per strain; primary y-axis). Significant differences in male ‘transition’ phenotypes across time and lineages are indicated by lowercase letters (linear model; Tukey post-hoc test; p < 0.05). Prevalence of (partheno-)sporophytes normalized to total number of gametophytes is shown as black line and text overlay (n = 5, mean ± SD). Significant increases in (partheno-)sporophyte numbers per time step within gametophyte lineages are indicated by asterisks (first asterisk, *T* test; following asterisks, linear model; Tukey post-hoc tests *** p < 0.001; ** p < 0.01; * p < 0.05; n.s., not significant). Significant differences in sporophyte prevalence across gametophyte lineages on day 40 are indicated by capital letters (linear model; Tukey post-hoc tests; p < 0.05). All scale bars = 50 µm. Note that for simplicity, all linear model results are collated in Table B in S1 Appendix. All photos were taken by D. Liesner. The data underlying this Figure can be found in S1 Data.

**Here, we use the kelp** *Laminaria pallida* **as a model system to investigate the regulation of developmental plasticity.** *L. pallida* occurs subtidally along the Atlantic coast of South Africa and Namibia, where the Benguela current fuels coastal upwelling of cold, nutrient-rich water [26]. Gametes of both sexes of *L. pallida* have recently been shown to initiate sporophyte development without fertilisation through parthenogenesis [27]. By combining transcriptomic analyses of sexual and asexual developmental pathways with population-level surveys in the wild, we characterize the molecular and ecological factors that govern these developmental fate decisions, with a particular focus on male parthenogenesis. We characterize core gene expression programs shared along development between asexual and sexual reproduction. Although both male and female gametes can develop parthenogenetically under laboratory conditions, extensive screening of natural populations revealed no evidence that asexual reproduction contributes substantially to population maintenance in the wild. We demonstrate that the presence of the opposite sex inhibits parthenogenetic development, suggesting that a diffusible signal mediates mate-dependent reproductive plasticity between asexual and sexual reproduction. Together with the reduced fitness of parthenogenetically derived sporophytes, this regulatory mechanism likely explains why asexual development remains rare in nature despite its developmental feasibility. These findings reveal an unexpected degree of developmental flexibility in a major marine lineage and provide new insights into how reproductive mode, developmental regulation, and ecological context interact to shape life-cycle evolution. More broadly, they advance our understanding of the mechanisms that maintain sexual reproduction despite the widespread latent capacity for asexual development and have implications for the conservation, restoration, and cultivation of kelp forests in a changing ocean.

## Results

### Parthenogenetic development is not restricted to female gametes

Parthenogenetic development of female gametes has been described in many kelp species [14], whereas the capacity of male gametes to initiate sporophyte development remains poorly characterized [23,27–29]. We therefore designed our experiment primarily to characterize male parthenogenesis, using clonal cultures of four male gametophytes and one female gametophyte as a reference and for sexual crosses. To compare sexual and asexual developmental trajectories, we monitored sporophyte formation in mixed and unmixed male and female gametophyte cultures (Table A in S1 Appendix; Fig 1A, 1B).

In unmixed cultures, we identified five distinct developmental stages: (1) vegetative gametophytes; (2) mature gametophytes bearing gametangia; (3) gametophytes after gamete release; (4) a transitional stage marked by the initiation of parthenosporophyte development; and (5) early parthenosporophyte formation, characterized by the emergence of two-dimensional blades (Fig 1B, 1C). Importantly, direct observations confirmed that male parthenosporophytes originated from individual sperm cells (Fig 1D; [27]), revealing no evidence for apogamy (i.e., development of sporophytes directly from vegetative gametophytes).

We next quantified the prevalence and timing of parthenogenetic development (Fig 1E). In all male lines, a subset of gametophytes underwent a developmental transition towards parthenosporophyte formation, characterized by the release of enlarged gametes from gametangia with an oogonium-like morphology (Fig 1D). This transition occurred during or shortly after gametangial maturation, between days 6 and 27 (Fig 1E). By day 40, the proportion of male gametophytes undergoing a parthenogenetic transition ranged from 4.22 ± 1.13% (Lp_M1) to 27.72 ± 1.71% (Lp_M3), with the latter not significantly different from the female rate (Tukey test, p = 0.3492). Importantly, despite the capacity for parthenogenesis in both sexes, sexual reproduction was substantially more efficient. Co-cultivation of male and female gametophytes produced more than twice as many sporophytes as parthenogenesis alone (Tukey test, p < 0.01), with an average of one sporophyte embryo generated per gametophyte.

Together, these results demonstrate that both female and male gametes can initiate sporophyte development in the absence of fertilisation. Although the frequency of male parthenogenesis varied among strains, some male lines produced parthenosporophytes at rates comparable to females. Sexual reproduction nevertheless remained substantially more effective in generating sporophytes.

### Parthenosporophytes exhibit abnormal development, endoreduplication, and reduced fitness

A subset of male and female parthenosporophytes, as well as zygotic sporophytes derived from crosses of the female with each male gametophyte line, were isolated and cultivated for further development (see Methods). After 31 weeks, clear morphological differences were observed between parthenosporophytes and zygotic sporophytes (Fig 2A). Sporophytes exhibited well-defined apical–basal thallus organisation, including differentiated holdfast, stipe, and blade regions. In contrast, parthenosporophytes displayed developmental abnormalities, such as malformed blades and occasionally incomplete differentiation of key tissues, particularly the holdfast and stipe. Extensive lateral growth often led to folds in the blade of parthenosporophytes. Despite these defects, parthenosporophytes could be grown for several months but remained significantly smaller compared to sexually derived sporophytes (Kruskal Wallis Χ^2^ = 27.78, p = 1.36*10^−7^; Fig 2B). PCR sex markers confirmed the presence of only one sex chromosome in asexually produced parthenosporophyte tissue (Fig 2C) and ploidy analysis by flow cytometry indicated that endoreduplication had occurred in all tested parthenosporophytes (S1 Fig). Despite three independent induction attempts, we did not observe the formation of reproductive structures in parthenosporophytes under our laboratory conditions.

**Fig 2:**
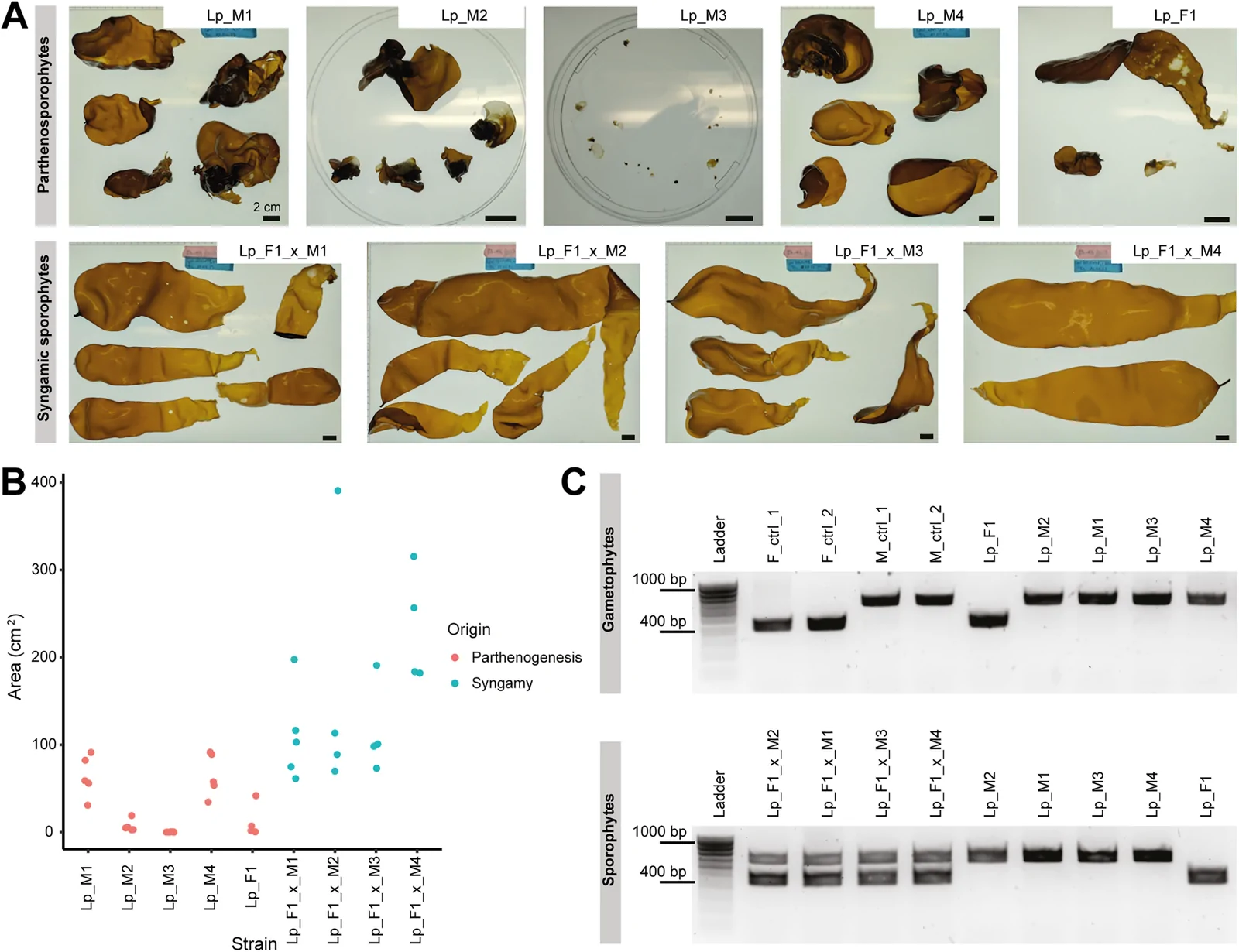
*Laminaria pallida* sporophyte characterisation. **(A)** Morphology of parthenogenetically and sexually derived sporophytes. Scale bar = 2 cm. **(B)** Sporophyte surface area of parthenosporophytes (red) and sexually derived sporophytes (blue). **(C)** Gel electrophoresis of sex-specific PCR markers indicating presence of a U-chromosome (450 bp) and a V-chromosome (700 bp) in gametophytes, parthenosporophytes and sexually derived sporophytes. All photos were taken by D. Liesner. The data underlying this Figure can be found in S1 Data. The uncropped gel image can be found in S1 Raw Images.

Together, these results indicate that parthenosporophytes may arise from both male and female gametes in kelp, that their development involves endoreduplication, and that they exhibit substantially reduced growth and developmental performance compared to zygotic sporophytes formed via sexual reproduction.

### Convergence of transcriptional signatures during parthenogenesis

To identify the molecular changes underlying the onset of parthenogenetic development and compare them with those associated with zygotic embryogenesis, we performed RNA sequencing (RNAseq) across defined developmental stages (Fig 1A, 1B). Principal component analysis (PCA) revealed distinct transcriptional signatures associated with sex and developmental stage (Fig 3A). Notably, although male gametophytes underwent pronounced transcriptomic changes upon reaching fertility, transcriptional trajectories from male and female parthenogenesis and sexual reproduction converged during the transition to sporophyte development.

**Fig 3:**
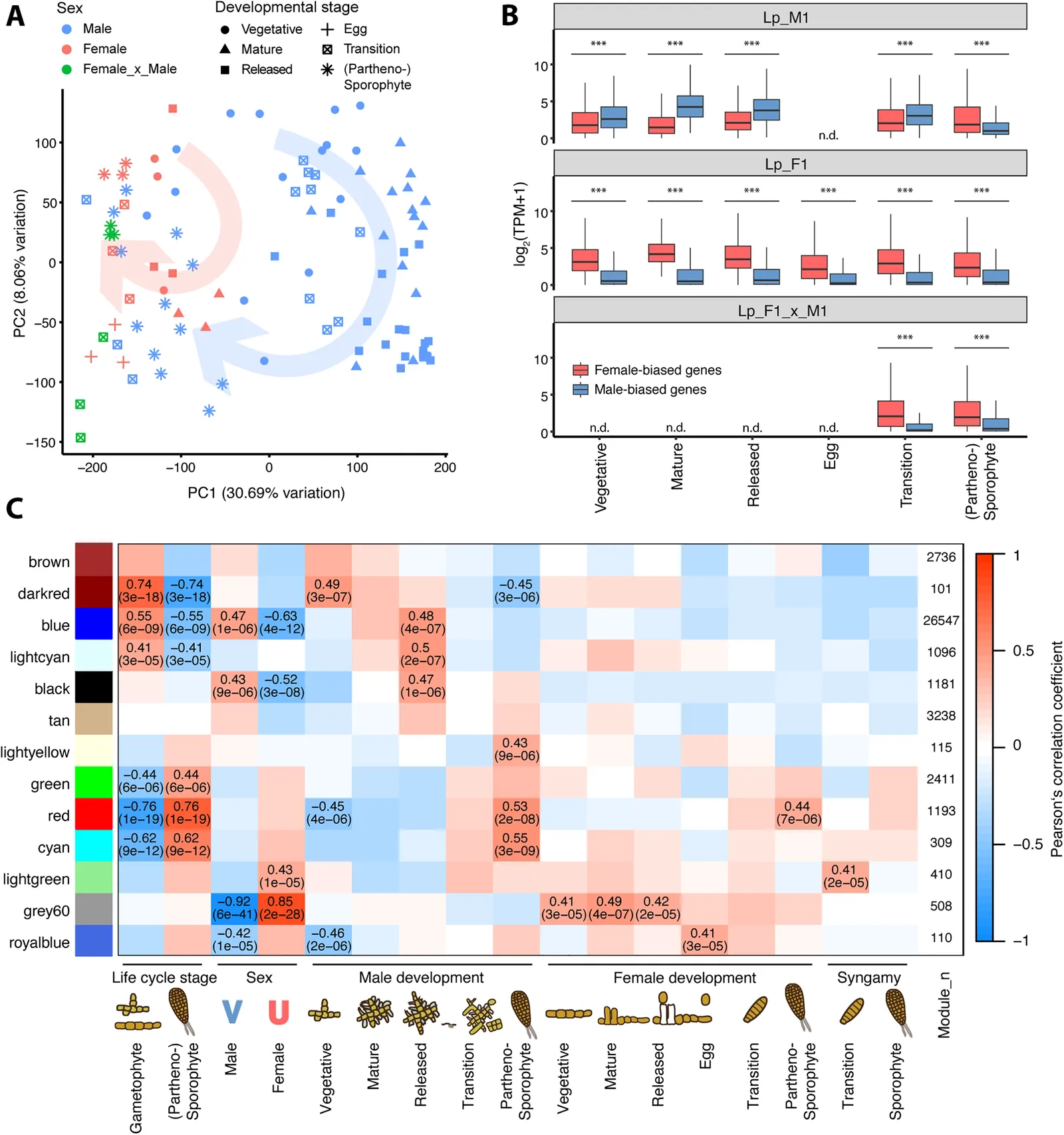
Gene expression patterns correlated to phenotype expression in *Laminaria pallida.* **(A)** Principal component analysis of RNA sequencing libraries with genetic sex indicated by colour and symbols distinguishing developmental stages (n = 98). Arrows indicate transcriptomic convergence along development towards sporophyte identity. **(B)** Sex-biased gene expression for male Lp_M1, female Lp_F1 and cross Lp_x_M1 across developmental stages; outliers are excluded for clarity; significant differences in sex-biased gene expression are indicated for each developmental stage (Wilcoxon rank sum test; *** p < 0.001); n.d., no data. **(C)** Relation of WGCNA module eigengenes to life cycle stages, genetic sex, and developmental stages of gametophyte and (partheno-)sporophyte development. For each module and trait, the heatmap indicates Pearson’s correlation coefficient **(R)**, and for |R| > 0.4, both R and p-value are displayed. Schematic drawings are partially modified from [30] published under CC BY 4.0. The data underlying this Figure can be found in S1 Data.

Sex-biased genes were identified for each strain as differentially expressed genes between male and female gametophytes during maturity (DESeq2; |log2FC| > 1; p < 0.001). While male-biased genes were dominantly expressed throughout development in all male strains, a significant reduction of male-biased gene expression occurred during parthenosporophyte development (Wilcoxon tests, p < 0.0001; Figs 3B; S3), leading to a transcriptomic de-masculinisation that resulted in expression profiles resembling those of female parthenosporophytes and zygotic sporophyte embryos.

Given the convergence of male parthenosporophyte and zygotic embryonic transcriptomes, we next examined the expression of conserved regulators of the gametophyte-to-sporophyte transition [17]. Two TALE homeodomain (HD) transcription factors that govern this developmental switch in the model brown alga *Ectocarpus*, SAMSARA and OUROBOROS [16,17], were identified in the *L. pallida* genome based on sequence homology (Table C in S1 Appendix; S4 Fig). Neither gene exhibited significant stage-specific expression (Wilcoxon rank sum tests, p > 0.05; no consistent differential expression, DESeq2). Instead, both were expressed throughout gametophyte and parthenosporophyte development.

We identified a core set of transcripts that was differentially expressed between parthenosporophytes and vegetative gametophytes of all lineages. Overall, 222 DEGs were consistently up-regulated and 84 transcripts were consistently down-regulated (S5 Fig; Table C in S1 Appendix). Homology search against the *Ectocarpus* genome revealed that transcripts related to known life cycle regulators were commonly up-regulated, namely three transcripts for *imm upregulated 13*, along with genes related to photosynthesis (light harvesting complex), cell wall metabolism (mannuronan C-5-epimerase), and brown algal viruses (EsV-1-7 domain).

A weighted gene co-expression network analysis (WGCNA) highlighted several co-expression modules related to differentiation between sexes and life cycle stages (Fig 3C). Notably, the largest module blue comprised 66.4% of all transcripts and was significantly associated with male gametophyte sexual development. This association, however, ceased during the transition stage and became significantly negative during parthenosporophyte development (R = −0.24; p = 0.02), indicating de-masculinisation of the transcriptome. The strongly enriched biological process gene ontology (GO) term “microtubule-based movement” indicates involvement in male gamete production (S6 Fig). Module grey60 was continuously associated with female development with a peak during maturity (Fig 3C) and was strongly enriched in the molecular function “DNA binding” (S7 Fig). Modules red and cyan were both significantly correlated to parthenosporophyte development (Fig 3C). In addition to metabolic processes related to photosynthesis and energy metabolism (“ATP metabolic process”, “lipid metabolic process”, “photosynthesis”, “light harvesting”), the enrichment of “peroxidase activity” and “oxidoreductase activity” indicates ROS signalling involved in sporophytic development (S8, S9 Figs). In module cyan, which is only significantly related to male but not female parthenogenesis (Fig 3C), the enrichment of “box H/ACA snoRNP assembly” and “protein folding” may be related to post-transcriptional and post-translational modifications specific to male parthenogenesis.

Together, these results indicate that male parthenogenesis is accompanied by transcriptome de-masculinisation, leading to convergence with female parthenogenesis and zygotic embryo development. Nevertheless, distinct expression signatures in life cycle–related genes and large co-expression modules suggest that these developmental trajectories remain molecularly distinct.

### No evidence for parthenogenetic reproduction in natural populations

To assess whether parthenogenesis has a relevant contribution to population structure and diversity in natural populations of *L. pallida*, we sampled sporophyte tissue from the populations of Paternoster, South Africa (ZAF) and Swakopmund, Namibia (NAM; S2A Fig; Table D in S1 Appendix). At each site, we employed a hierarchical sampling design spanning spatial scales from a few metres to several hundred metres. Specifically, we sampled at least 20 individuals from each of eight areas separated by < 5 m within each population (Figs 4A; S2B). Using 10 microsatellite markers (see Methods), we detected significant genetic differentiation between the ZAF and NAM populations (F_ST_ = 0.293, p = 0.050). Structure analysis identified K = 2 as the most likely number of genetic clusters following Evanno and colleagues [31] (S2D Fig), corresponding to the two sampled populations. No additional genetic substructure was detected within either population at K ≥ 3. Estimates of the inbreeding coefficient (F_IS_) did not differ significantly from expectations under random mating in either population (Fig 4B; Table 1), and F_IS_ did not differ between ZAF and NAM (F_1,18_ = 1.613, p = 0.22). Moreover, F_IS_ remained consistent across spatial scales within each population (S2C Fig). Together, these results indicate random mating and an absence of detectable fine-scale spatial genetic structure within the two populations.

**Table 1:** Genetic characteristics of the distinct *Laminaria pallida* populations of Paternoster, South Africa (ZAF) and Swakopmund, Namibia (NAM).

Population	n	N_a_	AR	H_e_	H_o_	F_IS_
ZAF	357	12.600 ± 2.766	11.264 ± 7.595	0.538 ± 0.097	0.526 ± 0.094	0.024 ± 0.031
NAM	166	4.800 ± 0.327	4.779 ± 0.975	0.456 ± 0.039	0.464 ± 0.046	−0.015 ± 0.023

n, number of individuals for which at least 8 markers amplified; N_a_, mean number of observed alleles; AR, allelic richness standardized for equal sample size (160 individuals); H_e_, expected heterozygosity; H_o_, observed heterozygosity; F_IS_, fixation index (inbreeding coefficient) of individuals with respect to local subpopulation. All parameters are expressed as means over all markers (*n* = 10) ± standard error.

**Fig 4:**
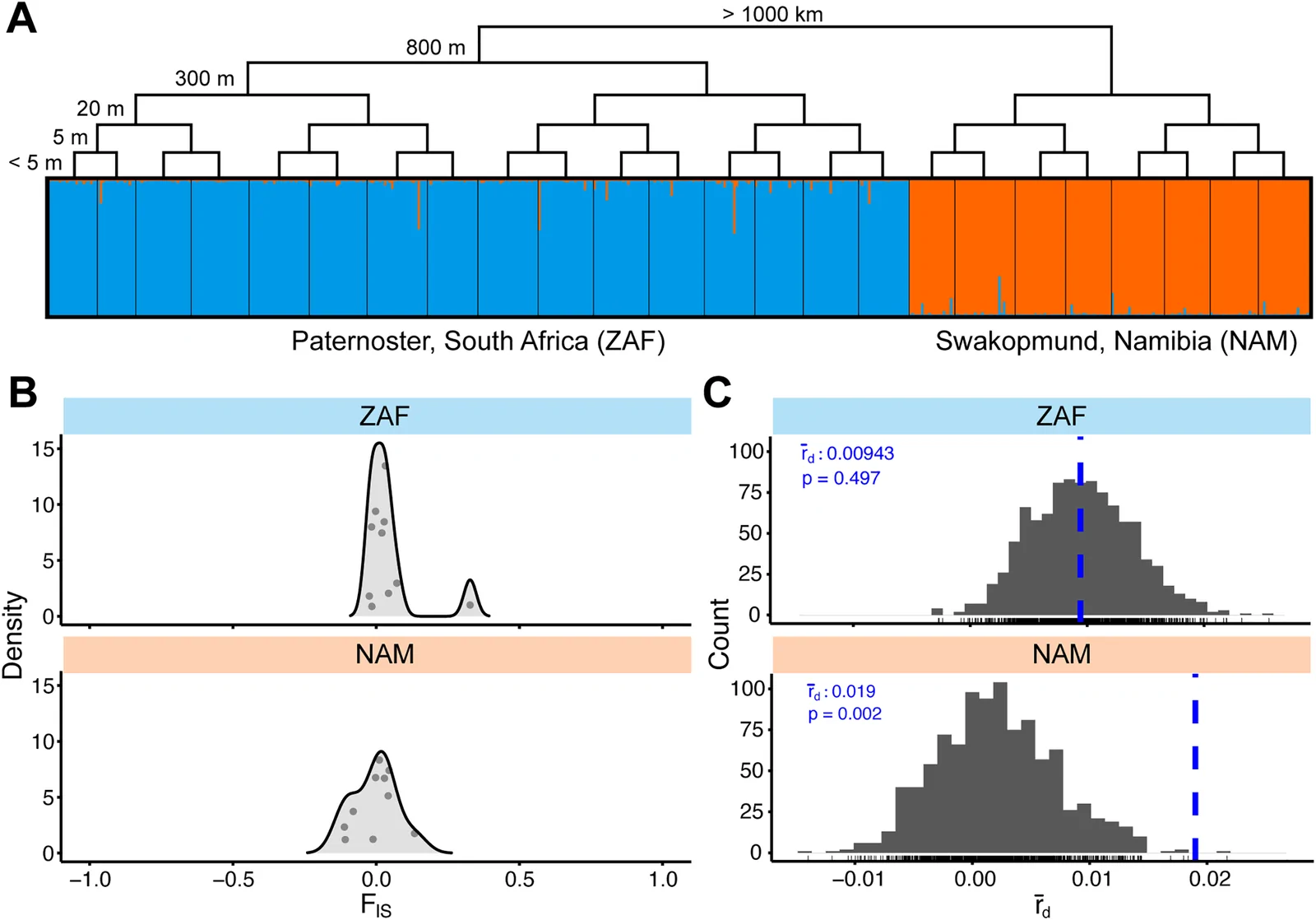
Population genetic characteristics of *Laminaria pallida* sampled at Paternoster, South Africa (ZAF; n = 357) and Swakopmund, Namibia (NAM; n = 166). **(A)** Structure bar plot obtained for K = 2 genetic clusters. Tree reflects the hierarchical sampling levels in decreasing order of magnitude. Individuals (vertical bars) were assigned probabilities of belonging to clusters (colors) based on differences in genetic variance. **(B)** Density plot of inbreeding coefficient F_IS_ for all microsatellite loci (n = 10) in ZAF and NAM. **(C)** Test for linkage disequilibrium by random permutations (n = 999) of the standardized index of association (r̄ d) shown as histograms and observed values shown by dashed blue lines for NAM and ZAF. The data underlying this Figure can be found in S1 Data.

The number of alleles N_a_ was significantly higher in ZAF than in NAM (ANOVA; F_1,18_ = 7.84, *p* = 0.012; Table 1), as was the allelic richness AR (ANOVA; F_1,18_ = 6.45, *p* = 0.021). However, neither expected heterozygosity H_e_ (ANOVA; F_1,18_ = 0.623, *p* = 0.44) nor observed heterozygosity H_o_ (F_1,18_ = 1.90, *p* = 0.185) differed significantly between populations. Additionally, we did not identify unambiguous evidence of parthenogenesis among the genotyped individuals, as no identical multilocus genotypes and no purely homozygous genotypes were identified. However, analysis of linkage disequilibrium based on the standardized multilocus index of association r̄ d revealed a significant deviation from random mating only in NAM but not in ZAF (Fig 4C). Together, these results indicate that asexual reproduction and clonality is not prevalent in nature.

To assess whether wild populations retain the capacity for asexual development despite its apparent absence in the field, we established 31 female and 28 male gametophyte cultures from a subset of collected sporophytes. Although bacterial contamination affected few samples and six isolates did not become reproductive, 92% of mature male and female gametophytes produced parthenosporophytes (Table E in S1 Appendix). These results indicate that the potential for clonal reproduction is maintained in both sexes in the natural populations examined, even though its rare realization among adult sporophytes suggests that asexual reproduction may be ecologically or competitively disadvantaged compared to sexual reproduction.

### Male parthenogenesis is suppressed in the presence of females

The analyses described above revealed no evidence of clonal reproduction in natural populations. This raises the question of how the capacity for parthenogenesis is maintained despite its apparent absence in natural populations. One possibility is that parthenogenesis provides reproductive assurance when mates are unavailable. To test this hypothesis, we examined whether the presence of a mating partner in the same culture medium separated by a 5 µm mesh influences the frequency of parthenogenetic development (see Methods). If so, parthenogenesis could function as a conditional reproductive strategy that is enhanced under mate limitation.

To test this hypothesis, we used the two male lines with the highest rates of parthenogenesis (Lp_M3 and Lp_M4; Fig 1E) together with the female line Lp_F1. In both male lines, the frequencies of transition phenotypes (Fig 5A) and parthenosporophyte development (Fig 5B) were significantly higher in the absence of a female partner. Females showed a similar response, producing more egg-stage gametophytes and parthenosporophytes when males were absent. Moreover, females released significantly more eggs in the absence than in the presence of males (2.61 ± 0.39 versus 1.75 ± 0.12 eggs per individual on day 6; *t* test, t = 4.677, p = 0.0062). While we cannot fully exclude that some spermatozoids may have passed the mesh (see gamete size in [30]), the abnormal morphology of sporophytes [20,32] and their lack of attachment to the maternal oogonium [22] strongly suggested parthenogenetic origin. Together, these results reveal that reproductive plasticity in *L. pallida* gametophytes is mediated by mate presence, suggesting that parthenogenesis and sexual reproduction are modulated by a yet undescribed mate-recognition mechanism.

**Fig 5:**
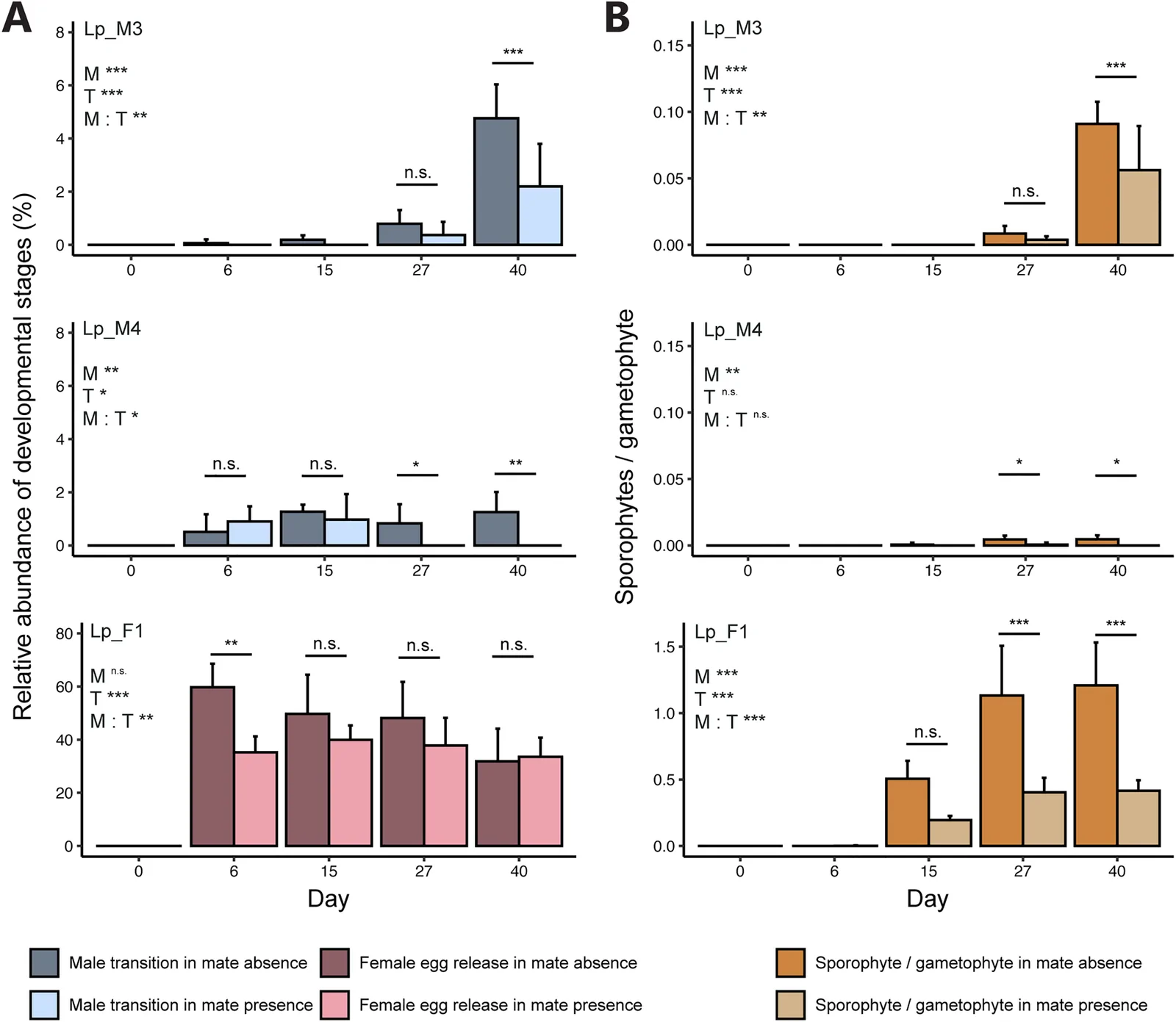
Effect of mate presence over time on the prevalence of parthenogenesis in *Laminaria pallida* gametophyte males (Lp_M3; Lp_M4) and females (Lp_F1) with respect to (A) gametophyte development and (B) sporophyte production. Statistical significance of the fixed factors mate presence (M), time (T), and their interaction (M: T), and results of pairwise comparisons (Tukey test) are summarized in the figure; *** p < 0.001; ** p < 0.01; * p < 0.05; n.s., not significant. Note that for simplicity, all linear model results are collated in Table F in S1 Appendix. The data underlying this Figure can be found in S1 Data.

## Discussion

### Mate-dependent regulation of parthenogenesis in kelps

Our results reveal an unexpectedly broad capacity for asexual reproduction in the kelp *Laminaria pallida*. While male parthenogenesis had been characterized in sibling gametophytes derived from one *L. pallida* sporophyte [27], our gametophytes collected from more than 25 sporophytes revealed that almost all lines tested were capable of parthenogenetic development under laboratory conditions. This trait is unlikely to be unique to *L. pallida*, as female parthenogenesis is widespread across kelps and male parthenogenesis has been reported from several genera worldwide [23,28,29,33], with growing evidence that asexual development may be more common than previously appreciated [27,34,35].

Despite this latent capacity, sexual reproduction clearly outperformed parthenogenesis in our experiments. Male parthenogenesis was slower and produced fewer offspring than female parthenogenesis, while sexually derived offspring were both more abundant and grew more rapidly than progeny produced through either male or female parthenogenesis. These findings indicate that sexual reproduction retains a substantial fitness advantage whenever mates are available.

Parthenogenesis is common among morphologically simpler brown algae [11,15], whereas more complex lineages such as fucoids and kelps are generally thought to rely on sporophyte-mediated forms of clonal propagation. In *Fucus vesiculosus*, for example, extensive single-sex clonal populations have become established in the Baltic Sea through fragmentation and reattachment [36,37]. Likewise, several kelp species propagate vegetatively through clonal blade production from creeping stolons [38–40]. Nevertheless, female parthenosporophytes have occasionally been reported to develop normally [41] and even reach reproductive maturity in natural populations [33], suggesting that gamete-derived asexual reproduction remains biologically relevant in kelps.

Yet, despite the universal capacity for parthenogenesis observed in our experiments, we found no evidence for clonal reproduction in natural populations of *L. pallida*. This apparent paradox is resolved by our mate-exclusion experiment, which demonstrates that parthenogenesis is modulated in response to mate availability. In both sexes, parthenogenetic development increased when mating partners were absent and declined when they were present. These findings point to a mate-recognition mechanism which mediates reproductive allocation between sexual and asexual reproduction. Rather than representing a competing reproductive strategy, parthenogenesis may therefore function as a conditional backup pathway that provides reproductive assurance when mates become limiting, while remaining largely masked under conditions that favour sexual reproduction.

### Shared developmental programs underpin parthenogenesis and embryogenesis

In *Ectocarpus*, the two TALE homeodomain transcription factors that trigger sporophyte development are present in both sexes, rendering life-cycle identity independent of syngamy [11,17,30]. Our results suggest that kelp gametophytes similarly possess the molecular machinery required to initiate the sporophyte program autonomously. However, unlike in *Ectocarpus* [16,17], both SAMSARA and OUROBOROS are continuously expressed throughout male and female development in *Laminaria pallida*. Whether SAMSARA and OUROBOROS are subject to regulation at other levels (e.g., post-transcriptional regulation [42]), or whether parthenogenetic development in *L. pallida* involves additional regulatory factors, remains to be investigated.

We identified core sets of genes and co-expression modules associated with sporophyte development during both parthenogenesis and zygotic embryogenesis. Among the genes shared across developmental trajectories were homologs of genes controlled by IMMEDIATE UPRIGHT (*imm upregulated 13*), which controls the initial asymmetric cell division during sporophyte development in *Ectocarpus* [43,44]. The enrichment of GO terms related to reactive oxygen species (ROS), which play important roles in embryogenesis in both plants and brown algae [45,46], suggests a potential involvement of ROS-related signalling in (partheno-)sporophyte development. Enrichment of photosynthesis and metabolism-related GO terms may reflect changes in energy metabolism associated with the morphological transition from filamentous to sheet-like growth, the latter potentially enabling more efficient photosynthesis [47]. These findings suggest that parthenogenetic and zygotic development recruit a common developmental program, despite their distinct origins. Because transitions between sexual and asexual reproduction can involve diverse genetic mechanisms [48], future studies will be required to identify the loci that regulate parthenogenesis in kelps.

Against this shared developmental background, male parthenogenesis is accompanied by a striking transcriptional reconfiguration. GO terms enriched in association with male parthenogenesis further point to a potential role for post-transcriptional regulation, which has recently been shown to contribute to the fine-tuning of life cycle stage identity in *Ectocarpus* [42]. We previously showed that reduced expression of male-biased genes results in a feminized gametophyte morphology, whereas male development depends on a complex network of sex-biased gene expression [30]. Consistent with this, male development in both *Macrocystis pyrifera* and *L. pallida* is associated with expression of a large fraction of the transcriptome [30]. Here, we show that parthenogenetic development is preceded by extensive de-masculinisation of the transcriptome following gamete release. Concomitantly, male gametes enlarge, lose the capacity for fusion, and acquire the ability to initiate parthenogenesis (see also [27]). Together, these observations suggest that male parthenogenesis involves a partial reprogramming away from a canonical gametic state, generating cells that may function primarily as clonal dispersal propagules rather than as gametes *sensu stricto*.

### Parthenogenesis as a conditional reproductive strategy

Despite the widespread occurrence of parthenogenesis in our laboratory experiments, we detected no unambiguous population-genetic signatures of asexual reproduction among 523 sporophytes sampled from Namibian and South African populations. This discrepancy suggests that parthenogenetically derived offspring contribute little to recruitment in natural populations. One explanation is that asexual offspring experience reduced fitness relative to sexually derived sporophytes, consistent with the delayed development and growth defects observed here. Indeed, fitness costs of facultative parthenogenesis on viability and fecundity have been documented in animals [49,50] and plants [51]. Population genetic modelling in giant kelp similarly predicts high early mortality among highly homozygous offspring produced through self-fertilisation [52].

Our experiments further indicate that parthenogenesis is regulated in response to mate availability. In both sexes, asexual development increased when mating partners were absent, suggesting that a mate-recognition mechanism mediates reproductive allocation between asexual and sexual developmental trajectories. In the female strain, the increased parthenogenesis in absence of males contrasts the higher reproductive success of fertilisation in the developmental time course experiment. Similar patterns have been proposed in giant kelp, where female egg production increases in the absence of males [53], and are consistent with the hypothesis that parthenogenesis functions as a form of reproductive assurance under mate limitation [54,55]. Such conditions may occur in sparsely populated habitats or during episodes of environmental stress, potentially facilitating persistence at distributional margins [10].

Evidence for such a role in natural populations, however, remains limited. We detected a weak but significant deviation from random mating in the Namibian population that could reflect cryptic clonality. Whether this pattern is related to local environmental conditions remains unclear. In summer, sea surface temperatures at Swakopmund reach 20°C and above (S2E Fig; see also [56]), a threshold that likely impairs growth and reproduction in *L. pallida* [32]. While sporophytes may reproduce year-round [57], gametophyte reproduction is optimal at 12°C [32], likely facilitating juvenile sporophyte recruitment in cold seasons [57] or upwelling periods. Despite warming of the northern Benguela region [58], however, the species’ northernmost population at Rocky Point, Namibia, has persisted since it was first documented in 1970 [59,60]. Whether the relative importance of asexual developmental pathways increases towards the distributional range limit warrants further investigation.

Together, these observations suggest that the evolutionary significance of parthenogenesis in kelps is likely to depend on ecological context. There is currently no evidence that parthenogenesis becomes more frequent at distributional range edges, despite reports of reproductive abnormalities in populations of *Laminaria digitata* near their warm range limit [61]. Moreover, parthenogenetically derived sporophytes appear less competitive than their sexually produced counterparts, owing to delayed development, irregular growth, and, in some species, reduced fertility [62]. Yet the evolutionary maintenance of this trait may not require substantial fitness benefits. Male gametophytes produce functional sperm [27], and in our experiments most parthenosporophytes developed only after the main period of gamete release. Thus, investment in asexual development occurs predominantly after opportunities for sexual reproduction have declined. Although we did not directly quantify the physiological costs of maintaining parthenogenetic capacity, this temporal separation may limit interference with sexual reproduction while retaining an alternative route to reproduction when mating fails.

In conclusion, our results reveal a widespread but tightly regulated capacity for parthenogenesis in kelps. Parthenogenetic and sexual development converge on a common sporophyte program, yet mate availability biases reproduction towards sex, potentially restricting the contribution of asexual offspring in natural populations. These findings suggest that parthenogenesis functions not as an alternative to sexual reproduction, but as a latent developmental pathway that may provide reproductive assurance when opportunities for mating are limited.

## Materials and methods

### Study material

Fertile sporophytes of *Laminaria pallida* had been collected on the shores of Kommetjie, South Africa, in 1999 (Lp_M1–Lp_M3), Swakopmund, Namibia, in 1993 (Lp_M4), and Paternoster, South Africa in 2022 (Lp_F1). Meiospores were released and clonal gametophyte isolates were prepared as described by [63]. All gametophytes were grown vegetatively in 50% Provasoli-enriched natural seawater (PES; [64]; iodine enrichment following [65]) under red light in a 14:10 h light:dark (L:D) cycle (MaxLED 500 RGBW, Paulmann, Springe, Germany) at 14°C in a thermostatic cabinet (TC 445 L, Lovibond, Dortmund, Germany). These conditions allow decade-long maintenance of vegetatively proliferating gametophyte cultures [63], while standardized common-garden conditions minimize environmental carry-over effects associated with the populations of origin [66,67]. Long-term vegetative maintenance may nevertheless allow the accumulation of somatic mutations or persistent non-genetic changes [68]. However, the cultures used here retained sexual functionality, and the strong parthenogenetic capacity independently observed in freshly collected natural material (Table E in S1 Appendix; [27]) indicates that parthenogenesis itself is not a consequence of long-term laboratory culture.

### Culture conditions

To induce gametophyte fertility, gametophyte tufts were carefully ground using mortar and pestle, sieved to obtain a size fraction of 50−100 µm, and sowed into 55 mm petri dishes into 50% PES to a final volume of 12 mL. After a recovery period of two days at reduced irradiance (5−10 µmol photons m^-2^ s^-1^), dishes were cultivated at 12°C in a 16:8 h L:D cycle at 20−30 µmol photons m^-2^ s^-1^ white light. Once per week, 5 mL of culture medium were replenished with 50% PES [32,63].

To confirm parthenogenetic and sexual reproduction in all gametophyte lines and to characterize the development of the resulting (partheno-)sporophytes, all lines were transferred to reproductive conditions in isolated and mixed (each male with Lp_F1) treatments. Sporophytes obtained from syngamy and asexually were isolated with tweezers at a size of ~1 mm. First, sporophytes were grown in 1L flasks with gentle aeration in a temperature-controlled chamber at MPI Tübingen at 14°C in a 12:12 h L:D cycle at 30–40 µmol photons m^-2^ s^-1^ in 100% PES. After four months, they were transferred to the Station Biologique de Roscoff, France, and grown at 13 ± 1°C in a 16:8 h L:D cycle at 30–40 µmol photons m^-2^ s^-1^ (T8 Lumilux 36W–865 Cool Daylight, Osram) in 100% PES. With increasing sporophyte size, culture volume was sequentially increased to 10L transparent round bottles (Nalgene). Medium was changed weekly, photos were taken on an LED table and sporophyte area was analysed using ImageJ Fiji [69].

### DNA isolation and sex marker PCR

To determine the genetic sex of gametophytes, parthenosporophytes and crossed sporophytes, genomic DNA was extracted from 50-100 mg tissue using the OmniPrep Genomic DNA isolation kit (G-Biosciences, St. Louis, USA) following the manufacturer’s instructions. For sex genotyping, we used the male marker ldig-hmg-g1-3–4 (F: ACGCACATCTTCCTTCGCTA, R: GCAAAAGGATCGGACACTGC; product 700 bp [70]) and female marker Lp_0170_3 (F: CAGCATTCTTGACGCACAGA, R: CCAGTGCCCCAAACAAGTAC; product 450 bp; this study). Sex-specific nucleotide sequences were identified by alignment of ancestral sex-linked gene sequences of *Ectocarpus* [71,72] to the *L. pallida* genome. PCR primers were then designed using Primer3.

PCR was performed using KAPA3G polymerase with both markers present in each reaction, on DNA extracted from two independent sets of female and male wild type gametophytes (control), the gametophyte strains tested in this study, and crossed and parthenogenetic sporophyte tissue. In the PCR, initial denaturation lasted for 3 min at 95°C, followed by 35 cycles of denaturation at 95°C for 20 sec, annealing at 61°C for 25 sec (first 10 cycles touchdown 66–61°C), elongation at 72°C for 15 sec, and a final elongation step at 72°C for 5 min.

### Genome sequencing, assembly and annotation

Genomic HMW DNA was isolated from *L. pallida* strains NAM93 female and KU1234 female (Table A in S1 Appendix) using the OmniPrep Genomic DNA Isolation kit (G-Biosciences, St. Louis, USA) following the manufacturer’s instructions. Sequencing libraries were prepared using Oxford Nanopore’s SQK-LSK110 kit and sequenced on a R9.4.1 MinION flowcell (FLO-MIN106). The initial basecalling was performed using Guppy v6.0.7 + c7819bc52 with the r9.4.1_450bps_hac configuration. A second basecalling was performed by Dorado v0.9.6 and the dna_r9.4.1_e8_sup@v3.6 protocol. An additional short-read sequencing library of *L. pallida* NAM93 male was prepared using a custom protocol for Tn5 tagmentation [30] and sequenced as 150 bp paired-end libraries on an Illumina NextSeq2000 at the Genome Core Facility at the MPI Tübingen Campus. A contamination analysis was performed to remove prokaryotic reads. The fastq reads were filtered for bacterial contamination by using a conjunction of Kraken2 v.2.1.2 [73] and blastn v2.9.0+ in combination with the NCBI nt database (download date 1 July 2022).

Oxford Nanopore long reads were assembled using Flye v2.9.2 [74] in default mode. Illumina short reads were individually assembled by Abyss v2.3.7 [75] (k = 48 kc = 3 B = 50G n = 5). ARCS v1.2.5 [76] (z = 1,500 m = 8–10,000 s = 70 c = 3 l = 3 a = 0.3) was used to scaffold the Nanopore Flye assembly with the Illumina Abyss assembly. Repeat soft-masking was performed by funannotate v1.8.17 [77] and default settings.

Structural gene annotation was performed by Helixer v0.3.6 [78] (--linage land_plant as the trained model most closely related to brown algae) and funannotate. AGAT agat_sp_merge_annotations.pl v1.5.1 [79] was used to merge the gene models of Helixer and funannotate. mRNA models spanning over >3 different gene models and models without RNAseq evidence were removed. TE filtered protein sequences of each mRNA model were extracted and analysed by InterProScan [80], mapped by Diamond [81] to the NCBI blast nr database, Araport11 [82] and the *Ectocarpus* sp7 annotation. Genome assembly and annotation statistics are collated in Table G in S1 Appendix.

### Developmental time course

Before the start of the experiment, gametophytes of all strains were treated with antibiotics for 14 days (Rifampicin 40 mg/L, Penicillin 100 mg/L, Streptomycin 25 mg/L, Chloramphenicol 5 mg/L, Kanamycin 50 mg/L, Cefotaxime 100 mg/L) with medium changes performed every 3−4 days. Filaments were sown to a target density of 500 gametophytes cm^-2^ and gametophyte fertility was induced as described above in 55 mm petri dishes (see Culture conditions). Additionally, five dishes containing both the male line Lp_M1 and the female line Lp_F1 were prepared at a target density of 250 gametophytes cm^-2^ per strain to obtain crossed sporophytes. Per strain, five replicate dishes were prepared (n = 5).

Development was assessed by microscopic counting (Axio Vert.A1, Zeiss, Jena, Germany) of least 300 individuals per replicate on days 0, 6, 15, 27, and 40 of the experiment, and assigning them to developmental stages (male: vegetative, mature, transition towards parthenogenesis; female: vegetative, oogonia, egg release, gametophyte with attached sporophyte). Additionally, the total number of emerging (partheno-)sporophytes was counted. Male gametophytes were categorized as transition phenotypes if they began to produce enlarged cells resembling oogonia, released enlarged immotile gametes, and/or produced sporophyte tissue from a gametophyte colony (see “Transition” category in Fig 1B, 1D). Biomass was collected at five developmental stages on days 1, 7, 16, 28, and 41: 1. Vegetative gametophytes; 2. Mature gametophytes with gametangia and first spontaneous gamete release; 3. Gametophytes after gamete release initiating vegetative growth; 4. Gametophytes during phenotype transition; in the male cultures this corresponds to reproductive variations as explained above, in the female wild type and crossing culture this corresponds to 2–5 cell (partheno-)sporophytes; 5. Blades of parthenogenetic and crossed sporophytes after syngamy (Fig 1A, 1B). Per replicate dish, 5–10 gametophyte filaments were collected using a drawn-out Pasteur pipette. Isolates were transferred in a 1 µL droplet of SW into 5 µL of lysis buffer (NEBNext Single Cell/Low Input RNA Library Prep Kit for Illumina), and processed according to the manufacturer’s instructions. A total of 99 cDNA libraries (*n* = 3) were sequenced on an Illumina NextSeq2000 as 150 bp paired-end reads.

### Gene expression analysis

Reads from 99 low-input RNAseq libraries were mapped to the *L. pallida* genome using HiSat2 v2.2.1 [83] (--very-sensitive --new-summary -q --max-intronlen 25,000). Gene read abundances were summarized using featureCounts v2.1.1 [84] (-p --countReadPairs -C -s 0 -M -O --largestOverlap –primary). Transcript abundances were calculated as transcript per million (TPM) and normalized as log2(TPM + 1). Using a per-library expression threshold of TPM values > 5th percentile, excluding genes with null expression, we identified all 39,955 mRNA transcripts as expressed in at least one library. Therefore, all mRNA transcripts were included in the downstream analyses.

Differential gene expression was analysed using DESeq2 v1.46.0 [85]. The significance threshold for differentially expressed genes was considered at log2 fold-change |log2FC| > 1 and FDR-adjusted padj < 0.001. Sex-biased genes were defined for each strain as the set of DEGs between the respective male and the female gametophyte Lp_F1 during maturity. For Lp_F1, Lp_M1 was chosen as the male comparison. Differences in normalized expression between female- and male-biased genes along development were analysed using pairwise Wilcoxon rank sum tests.

A weighted gene co-expression network analysis was conducted using R package WGCNA v1.73 [86] using log_2_(TPM + 1) normalized data as input. Genes with null expression were excluded from the analysis, and one library was excluded as an outlier due to lack of sample clustering, leaving 98 low-input RNAseq libraries for the analysis. A signed network was constructed using a biweight midcorrelation (‘‘bicor’’), a soft thresholding power of 9, and maximum portion of outliers of 0.05. To obtain a reasonable number of gene co-expression modules with sufficient size for statistical analyses, modules were constructed with a minimum size of 30 transcripts and were merged to a correlation threshold of 0.75. We correlated gene co-expression module eigengenes to traits related to life cycle stage (gametophyte, sporophyte), genetic sex (female, male), and developmental stages for each gametophyte sex and zygotic embryo development (see Fig 3C). Significant enrichment of gene ontology (GO) terms within co-expression modules and differentially expressed gene sets was analysed using Fisher’s exact test (p < 0.05) within R package topGO version 2.58.0 [87].

### Field sample collection

To identify potential signals of clonality in field populations of sporophytes, we collected sporophyte tissue from *L. pallida* populations close to Swakopmund, Namibia (NAM, −22.699571 N, 14.520699 E) and Paternoster, South Africa (ZAF, −32.81789 N, 17.85747 E; and −32.81288 N, 17.86865 E; S2A Fig) in February and March 2022. At each location, we applied a hierarchical sampling design considering areas separated by three spatial levels: hundreds of metres (~300 m), tens of metres (~20 m), and few metres (~5 m), in which we collected samples from at least 20 individuals in each of eight areas (~2 m²) within the same population (S2B Fig). When increasing the sampled area to ensure a sufficient number of samples (up to approx. 25 m²), distances between areas were increased, too, to maintain a hierarchical design. In NAM, four areas were adjusted to transects of 15–30 m length to accommodate for the shore geography and population patchiness. In ZAF, 393 sporophytes were sampled, and 180 in NAM. Using cork borers, tissue discs of approx. 20 mm diameter were cut above the meristematic region of adult individuals whereas smaller individuals (< 15 cm) were removed whole. Samples were collected in ziplock bags and transported in a cool box. All tissue discs were wiped clean and rinsed in distilled water. Vegetative discs were then dried and preserved in ziplock bags filled with silica gel. Fertile sorus tissue was collected in centrifuge tubes filled with seawater and glass slides as settling substrate. Following spore release in the dark, monoclonal gametophyte isolates were produced [63] and maintained as described above.

#### DNA extraction and microsatellite amplification.

DNA was extracted from dried tissue using the Nucleospin 96 Plant II kit (Macherey-Nagel, Düren, Germany) according to the manufacturer’s instructions using buffer PL1 for cell lysis. For each individual, 15 microsatellite loci were amplified to generate multi-locus genotypes. PCR reactions were carried out in 10 µL reaction volume following the protocol of Guzinski et al. [88], in which one of four fluorescent labels is added for multiplex sequencing following DNA amplification. Three to four samples were pooled, added to loading buffer including a size standard (SM594; [89]) and subsequently sequenced in an ABI 3130 XL capillary sequencer (Applied Biosystems, Waltham, USA) at Genomer, Plateforme génomique, Biological Station of Roscoff, France. Alleles were scored manually using GeneMapper v4.0 (Applied Biosystems, Waltham, USA).

Ten markers were retained for the analysis of genetic structure and diversity. Three markers had been developed for *Laminaria digitata* (CN466672 [90]; Ld19_025, Ld19_067 [91]) and seven for *Laminaria ochroleuca* (Lo5-8, LoIVVIV_15, LoIVVIV_16, LoIVVIV_24, LoIVVIV_26, LoIVVIV_27, LoIVVIV_28 [92]). Due to faulty amplification or multiple peaks, alleles of the additional markers Ld2_148, Ld19_034, Ld19_038 [91], and Ld2_357 [93] were not scored. Due to extreme polymorphism of locus LoIVVIV_23 [92] (114 alleles), this locus was removed from the analysis. Three loci showed evidence for a null allele according to the Oosterhout estimation in Micro-checker v2.2 (ZAF, Ld_19_025, frequency 0.0614; LoIVVIV_26, freq. 0.0398; NAM, LoIVVIV_24, freq. 0.0749; Table H in S1 Appendix). However, estimated null allele frequencies were low (< 8%; [94]) and were not consistent across populations. We therefore retained these loci, as excluding three markers would substantially reduce the information content of the dataset. We acknowledge that null alleles can lead to underestimation of heterozygosity and an inflation of estimates of genetic differentiation; however, given their low and inconsistent frequencies, and considering that only two populations were compared, we expect their potential bias to have a limited effect on the population-level patterns examined here. All markers were considered independent as no significant linkage disequilibrium could be observed within the populations (Table I in S1 Appendix). The number of alleles per locus ranged between five (Lo5-8) and 31 (LoIVVIV_24). Only samples in which at least 8 markers amplified were retained for analysis.

#### Genetic diversity.

The presence of null alleles was tested using the software Micro-checker v2.2 [95]. Standard measures of genetic diversity were calculated for each of the eight areas sampled per location as number of alleles per locus (N_a_), unbiased expected heterozygosity (H_e_), observed heterozygosity (H_o_), and number of private alleles (P_a_) using GenAlEx v6.5 [96]. Significant differences of diversity indices between populations were analysed by analysis of variance (ANOVA) in R v4.2.1 [97] using package nlme [98] using microsatellite loci as replicates. Allelic richness, linkage disequilibrium between pairs of loci and estimates of deviation from random mating (F_IS_) according to [99] were calculated using FSTAT v2.9.3 [100]. F_IS_ was computed within each level of sampling hierarchy [101] for all populations, and for the populations of ZAF and NAM separately. We also report single locus F_IS_ values for ZAF and NAM as suggested by [102,103] since the distribution of their values can be a signature of clonality. Significance of linkage disequilibrium was assessed using 1800 permutations, and significant deviation of F_IS_ from 0 was assessed using a *t* test (n = 10 loci). The standardized index of association between loci (r̄ d; [104,105]) was computed using Poppr v2.9.7 [106] and 999 permutations were applied to test whether loci were randomly associated. The distribution of clonal membership (Pareto ß [107]) could not be computed due to lack of repeated multilocus genotypes.

#### Population structure.

Genetic substructure was analysed using several methods. Genetic differentiation between ZAF and NAM was calculated as pairwise fixation index F_ST_ [99] using FSTAT v2.9.3 with 20 permutations (F_ST_ between quadrats with 5,520 permutations). Structure v2.3.4 [108] was run with 20 iterations, a burn-in period of 10^5^ and a Markov chain Monte Carlo of 10^6^. Structure Harvester Web v0.6.94 was used to identify the most likely number of clusters K [109]. Structure analysis was repeated on the identified subpopulations to identify potential hierarchical structure. Replicates of Structure runs were combined using CLUMPP v1.1.2 [110] and bar plots were created with Distruct v1.1 [111], using CLUMPAK Web v1.1 [112].

### Mate exclusion experiment

To assess whether mate presence has an effect on asexual reproduction of gametophytes, we prepared 90 mm petri dishes by fixing a fitted 5 µm nylon filter (Spectra/Mesh, Spectrum Labs, Rancho Dominguez, USA) across the diameter using food-grade duplication silicone (Wagnersil 32N, Wagner Dental GmbH & Co. KG, Hückelhoven, Germany). This allowed us to follow male gametophyte development in the presence of females without confounding the observations by presence of sporophytes or by false positive categorisations of female gametophytes as transitioning males. To this end, we seeded five replicate dishes of each male strain Lp_M1 – Lp_M4, and the female wild type Lp_F1 on either side of the mesh as a negative control (absence of mate) using the method described above. As a mate presence treatment, we seeded further five replicate dishes of the four male strains only on one side, while the other side was seeded with the female wild type. Gametophyte filaments were sown to a target density of 500 gametophytes cm^-2^ in 30 mL 50% PES. Once per week, 20 mL of culture medium were replenished with 50% PES.

After a two-day period of recovery and settlement, dishes were placed on a shaker (Heidolph Promax 2020) at 50 rpm to ensure the medium traversed the filter (tested in advance using a dye, medium was mixed in < 1 h). Apart from this, the experiment was conducted as above (see Culture conditions and Developmental time course) and development of male gametophytes was assessed in the presence and absence of females. In the dishes with Lp_M1 and Lp_F1 both males and females were assessed to also investigate the effects of male mate presence on female development.

### Statistical analysis

Developmental count data from the time course and mate exclusion experiments were analysed in R v4.2.1 [97] using linear mixed effects models (package nlme v3.1-167 [98]) and post-hoc tests were conducted using emmeans v1.10.7 [113]. Data of male transition phenotypes across strains and time were subjected to the variance-stabilising square-root transformation to account for variance heterogeneity in the model residuals [114]. To reduce inflation with zero-values in the linear models, the first time point where the respective phenotype (transition, sporophyte, egg release) occurred significantly was determined by a *t* test. The models were then fitted on all data obtained from this time point onwards. Plots were constructed using ggplot2 [115].

*Developmental time course.* Percentages of male transition phenotypes were modelled in response to the fixed interactive factors strain and time with random slopes assigned to each replicate to account for the repeated measurement design. Fractions of sporophytes per gametophyte were modelled for each strain and cross in response to time, with random slopes assigned to each replicate. Final number of sporophytes per gametophyte on day 40 was modelled in response to strain.

*Mate exclusion experiment.* Percentages of male transition phenotypes and female gametophytes with released eggs were modelled for each strain in response to the fixed interactive factors mate presence and time with random slopes assigned to each replicate to account for the repeated measurement design. Fraction of sporophytes per gametophyte were modelled for each strain and cross in response to the interaction of partner presence and time, with random slopes assigned to each replicate. The number of female eggs released per gametophyte in response to partner presence was compared using a *t* test.

## Supporting information

S1 Fig Results of flow cytometry analysis to estimate DNA content of *L. pallida* gametophyte (GA), partheno-sporophyte (pSP) and sporophyte (SP) material of five strains (males M1, M2, M3, M4; female F1; cross F1_x_M1, F1_x_M2). Plots show density of fluorescence signals (i.e., number of nuclei) as a proxy for DNA content (DNA-H). Peaks between DNA-H of 1.0–1.5 were interpreted as 1C (see presumably haploid gametophyte samples GA), peaks from 1.8–2.7 as 2C (multiplication factor 1.8). Peaks may vary in position due to variation between independent measurements. We interpret multiple peaks as mitosis in actively dividing cells. The data underlying this Figure can be found in S1 Data.
(TIF)

S2 Fig Wild populations of *Laminaria pallida* investigated in this study. **(A)** Map depicting the location of Swakopmund, Namibia (NAM) and Paternoster, South Africa (ZAF) based on the Natural Earth Admin 0 – Countries shapefile (https://www.naturalearthdata.com/downloads/10m-cultural-vectors/10m-admin-0-countries/). **(B)** Schematic hierarchical sampling design applied at the two populations. **(C)** Inbreeding coefficient F_IS_ over spatial scales in the hierarchical sampling design for ZAF, NAM, and both populations (mean ± 95% CI). **(D)** ΔK (Evanno et al., 2005) plotted against number of genetic clusters (K), for K = 2 to K = 9 obtained with Structure Harvester. **(E)** Variation of daily sea surface temperatures for NAM and ZAF from 2013 to 2022 (10 years before time of sampling) obtained from the E.U. Copernicus Marine Service (https://doi.org/10.48670/moi-00165) with daily means shown as a solid line and shading indicating standard error. The data underlying this Figure can be found in S1 Data.
(TIF)

S3 Fig Expression of sex-biased genes along development for four male (M1, M2, M3, M4) and one female strain (F1) of *Laminaria pallida* and their cross (F1_x_M1). Significant differences between male- and female-biased gene expression are indicated with asterisks (Wilcoxon rank sum tests; ** p < 0.01; ***, p < 0.001); n.d., no data. The data underlying this Figure can be found in S1 Data.
(TIF)

S4 Fig Expression of life-cycle related TALE homeodomain transcription factors (A) SAMSARA (1 transcript in n = 3 replicates) and (B) OUROBOROS (4 transcripts in n = 3 replicates) along development for four male (M1, M2, M3, M4) and one female strain (F1) of *Laminaria pallida* and their cross (F1_x_M1); n.d., no data. The data underlying this Figure can be found in S1 Data.
(TIF)

S5 Fig Intersection of (A) up-regulated and (B) down-regulated differentially expressed genes (DEGs) between (partheno-)sporophytes and their corresponding gametophyte for four male (M1, M2, M3, M4) and one female strain (F1) of *Laminaria pallida* and their cross (F1_x_M1). Only the 15 largest intersections are shown. The data underlying this Figure can be found in S1 Data.
(TIF)

S6 Fig Enriched gene ontology (GO) terms for (A) biological process, (B) molecular function, (C) cellular component in module “blue” of weighted gene co-expression network analysis of *Laminaria pallida* development. A maximum of 10 most enriched terms is displayed in dotplots sorted by significance (Fisher’s exact test). The data underlying this Figure can be found in S1 Data.
(TIF)

S7 Fig Enriched gene ontology (GO) terms for (A) biological process, (B) molecular function, (C) cellular component in module “grey60” of weighted gene co-expression network analysis of *Laminaria pallida* development. A maximum of 10 most enriched terms is displayed in dotplots sorted by significance (Fisher’s exact test). The data underlying this Figure can be found in S1 Data.
(TIF)

S8 Fig Enriched gene ontology (GO) terms for (A) biological process, (B) molecular function, (C) cellular component in module “red” of weighted gene co-expression network analysis of *Laminaria pallida* development. A maximum of 10 most enriched terms is displayed in dotplots sorted by significance (Fisher’s exact test). The data underlying this Figure can be found in S1 Data.
(TIF)

S9 Fig Enriched gene ontology (GO) terms for (A) biological process, (B) molecular function, (C) cellular component in module “cyan” of weighted gene co-expression network analysis of *Laminaria pallida* development. A maximum of 10 most enriched terms is displayed in dotplots sorted by significance (Fisher’s exact test). The data underlying this Figure can be found in S1 Data.
(TIF)

S1 Appendix Table A. Description of *L. pallida* gametophyte lines used in the experiment. **Table B.** Results of linear mixed effects models on responses during the developmental time course of *Laminaria pallida*. **Table C.** Expression data and functional annotation of all transcripts of *Laminaria pallida*. Functional annotation based on blastx against the *Ectocarpus* Ec32 genome, nr and Araport11 databases, gene ontology (GO) terms, normalized read counts (transcripts per million, TPM) strains Lp_M1, Lp_M2, Lp_M3, Lp_M4, Lp_F1 and cross Lp_F1_x_M1, along developmental stages vegetative (veg), mature (gam), released (rel), transition (tra) and sporophyte blade (bla), shared sporophyte-related genes based on DESeq2 results (sporophyte-gametophyte comparison), gene affiliation to gene co-expression modules, assignment to gene co-expression modules based on weighted gene co-expression analysis (WGCNA). **Table D.** Exact locations of the *L. pallida* sampling sites. **Table E.** Obervations of parthenogenesis during prolonged cultivation of unialgal gametophyte cultures isolated from *L. pallida* field material. **Table F.** Results of linear mixed effects models on responses during the mate exclusion experiment on *Laminaria pallida* gametophytes. **Table G.** *Laminaria pallida* genome assembly and annotation statistics. **Table H.** Null allele estimation (Oosterhout estimate within micro-checker) for each microsatellite locus for populations of *Laminaria pallida* from Swakopmund, Namibia (NAM) and Paternoster, South Africa (ZAF). Significant estimates are highlighted in bold font. **Table I.** Linkage disequilibrium estimation within FSTAT for each pair of microsatellite loci for populations of *Laminaria pallida* from Swakopmund, Namibia (NAM), Paternoster, South Africa (ZAF), and both populations. D’ values and p-values are given. Adjusted p-value based on 1800 permutations is 0.00056.
(XLSX)

S1 Data File containing all numerical values underlying the summary data displayed in each Figure. (XLSX)

S1 Raw Images Original uncropped image of the gel results presented in Fig 2C. (TIF)

## Abbreviations

GO: gene ontology
HD: homeodomain
PCA: Principal component analysis
PES: Provasoli-enriched natural seawater
ROS: reactive oxygen species
RNAseq: RNA sequencing
TPM: transcript per million
WGCNA: weighted gene co-expression network analysis
