# Supplementary material for: Life-cycle plasticity enables conditional asexual reproduction in a kelp
Source: PLoS Biol. 2026 Nov 12;24(11):e3004065. doi: 10.1371/journal.pbio.3004065 (PMC13759113; doi:10.1371/journal.pbio.3004065)
Supplement: Supplementary file 1 [file pbio.3004065.s015.docx]

Dear Susana,

Thank you for your patience while your manuscript "Life-cycle plasticity enables conditional asexual reproduction in a kelp" was peer-reviewed at PLOS Biology. It has now been evaluated by the PLOS Biology editors, an Academic Editor with relevant expertise, and by four independent reviewers. 

You'll see that reviewer #1 is impressed by the study, but raises a number of issues. Specifically, s/he identifies two points on which they think the evidence is insufficient; one is the case for bidirectional mate-recognition (whether this is active or passive), and one is the extrapolation from just two natural populations. Reviewer #2 also raises a number of issues where they believe that you can’t rule out alternative explanations, and more experimental data may be needed (his/her points 2, 3 and 4, regarding interspecific regulatory differences and bidirectional mate signalling). Reviewers #3 and #4 are both very positive, and only have textual requests.

In light of the reviews, which you will find at the end of this email, we would like to invite you to revise the work to thoroughly address the reviewers' reports.

Given the extent of revision needed, we cannot make a decision about publication until we have seen the revised manuscript and your response to the reviewers' comments. Your revised manuscript is likely to be sent for further evaluation by all or a subset of the reviewers.

In addition to these revisions, you will need to complete some formatting changes, which you will receive in a follow up email. A member of our team will be in touch with a set of requests shortly.

We expect to receive your revised manuscript within 3 months. Please email us (plosbiology@plos.org) if you have any questions or concerns, or would like to request an extension. 

At this stage, your manuscript remains formally under active consideration at our journal; please notify us by email if you do not intend to submit a revision so that we may withdraw it.

**IMPORTANT - SUBMITTING YOUR REVISION**

Your revisions should address the specific points made by each reviewer. Please submit the following files along with your revised manuscript:

1. A 'Response to Reviewers' file - this should detail your responses to the editorial requests, present a point-by-point response to all of the reviewers' comments, and indicate the changes made to the manuscript. 
*NOTE: In your point-by-point response to the reviewers, please provide the full context of each review. Do not selectively quote paragraphs or sentences to reply to. The entire set of reviewer comments should be present in full and each specific point should be responded to individually, point by point.
You should also cite any additional relevant literature that has been published since the original submission and mention any additional citations in your response. 

2. In addition to a clean copy of the manuscript, please also upload a 'track-changes' version of your manuscript that specifies the edits made. This should be uploaded as a "Revised Article with Changes Highlighted" file type. 

*Re-submission Checklist*
When you are ready to resubmit your revised manuscript, please refer to this re-submission checklist: https://plos.io/Biology_Checklist

To submit a revised version of your manuscript, please go to https://www.editorialmanager.com/pbiology/ and log in as an Author. Click the link labelled 'Submissions Needing Revision' where you will find your submission record. 

Please make sure to read the following important policies and guidelines while preparing your revision:

*Published Peer Review*
Please note while forming your response, if your article is accepted, you may have the opportunity to make the peer review history publicly available. The record will include editor decision letters (with reviews) and your responses to reviewer comments. If eligible, we will contact you to opt in or out. Please see here for more details:
https://blogs.plos.org/plos/2019/05/plos-journals-now-open-for-published-peer-review/

*PLOS Data Policy*
Please note that as a condition of publication PLOS' data policy (http://journals.plos.org/plosbiology/s/data-availability) requires that you make available all data used to draw the conclusions arrived at in your manuscript. If you have not already done so, you must include any data used in your manuscript either in appropriate repositories, within the body of the manuscript, or as supporting information (N.B. this includes any numerical values that were used to generate graphs, histograms etc.). For an example see here: http://www.plosbiology.org/article/info%3Adoi%2F10.1371%2Fjournal.pbio.1001908#s5 

*Blot and Gel Data Policy*
We require the original, uncropped and minimally adjusted images supporting all blot and gel results reported in an article's figures or Supporting Information files. We will require these files before a manuscript can be accepted so please prepare them now, if you have not already uploaded them. Please carefully read our guidelines for how to prepare and upload this data: https://journals.plos.org/plosbiology/s/figures#loc-blot-and-gel-reporting-requirements 

*Protocols deposition*
To enhance the reproducibility of your results, we recommend that if applicable you deposit your laboratory protocols in protocols.io, where a protocol can be assigned its own identifier (DOI) such that it can be cited independently in the future. Additionally, PLOS ONE offers an option for publishing peer-reviewed Lab Protocol articles, which describe protocols hosted on protocols.io. Read more information on sharing protocols at https://plos.org/protocols?utm_medium=editorial-email&utm_source=authorletters&utm_campaign=protocols

Thank you again for your submission to our journal. We hope that our editorial process has been constructive thus far, and we welcome your feedback at any time. Please don't hesitate to contact us if you have any questions or comments.

Sincerely,

Roli


Roland Roberts, PhD
Senior Editor
PLOS Biology
rroberts@plos.org

------------------------------------

Dear Roli

We thank the reviewers for their positive and constructive feedback on our manuscript. We responded to all requests and made the according changes in the manuscript. Our responses are highlighted in blue. Line numbers in our responses relate to the manuscript version without tracked changes.

General changes to the manuscript include an increased focus on male parthenogenesis to highlight the principal novelty of our study while reducing generalizations (re: reviewer 1), a more nuanced discussion of potential mechanisms involved in the proposed mate recognition (re: reviewers 1 and 2), and a reduced focus on parthenosporophyte fitness (re: reviewers 1 and 2). We also implemented all additional clarifications in response to reviewers 3 and 4.

REVIEWERS' COMMENTS:

Reviewer #1:

The paper by Liesner and colleagues presents a thorough investigation of parthenogenesis versus sexual reproduction in a species of brown alga.

Overall, I have no major concerns regarding the methodologies employed or the associated statistical analyses. The study is well written, clear, and relatively accessible even to readers who are not specialists in algal biology. Several of the results are both exciting and compelling, others required additional support (in my opinion).

My main concerns relate to the strength of some of the conclusions and whether they should be toned down or revised to better reflect the available evidence. A second concern is whether findings from a single algal species can reasonably be interpreted as representing a broader or universal pattern across lineages exhibiting both parthenogenetic and sexual reproductive modes.

Specific comments are provided below.

1. Novelty and broader significance:

One of the strongest and most novel findings of this study is the demonstration that male as well as female gametes possess autonomous sporophyte developmental capacity. Female gamete parthenogenesis has already been reported in several brown algal systems; therefore, the demonstration of male gamete-derived sporophyte development, together with the identification of endoreduplication as a mechanism underlying this developmental transition, represents an important advance.

However, the authors should be cautious when emphasizing the broader novelty of the finding that parthenogenetic sporophytes exhibit fitness disadvantages relative to sexually derived diploid sporophytes. Similar fitness costs associated with parthenogenesis have been documented in a variety of other taxa. Consequently, the principal novelty of the study lies not in demonstrating fitness disadvantages per se, but rather in revealing these patterns within this algal system and linking them to the underlying developmental and genomic mechanisms.

**Re:** We thank the reviewer for their thoughtful and positive comments. We agree that the reduced fitness of parthenogens is not a central aspect of our study and have reduced the emphasis on this point throughout the manuscript while highlighting our focus on the developmental transitions in male parthenogenesis.

**Ln. 20:** “We show that asexual reproduction is associated with endoreduplication and reduced fitness, while it is retained but rarely expressed in two natural populations.”

**Ln. 77:** “Here, we use the kelp *Laminaria pallida* as a model system to investigate the regulation of developmental plasticity. *L. pallida* occurs subtidally along the Atlantic coast of South Africa and Namibia, where the Benguela current fuels coastal upwelling of cold, nutrient-rich water [26]. Gametes of both sexes of *L. pallida* have recently been shown to initiate sporophyte development without fertilisation through parthenogenesis [27]. By combining transcriptomic analyses of sexual and asexual developmental pathways with population-level surveys in the wild, we characterize the molecular and ecological factors that govern these developmental fate decisions, with a particular focus on male parthenogenesis.”

**Ln. 384:** “Indeed, fitness costs of facultative parthenogenesis on viability and fecundity have been documented in animals [49,50] and plants [51].”

2. Developmental reprogramming and mate-recognition mechanisms:

The observation that male gamete-derived developmental programs become transcriptionally "less male" and shift toward a female-like or zygotic state provides a compelling mechanistic insight into developmental reprogramming. This finding has potentially broad implications for our understanding of developmental plasticity, sex-specific gene regulation, and the evolution of asexual reproduction.
However, I am less convinced by the strength of the evidence supporting the conclusion that there is "a bidirectional mate-recognition mechanism that promotes sexual reproduction while actively suppressing parthenogenesis."

The observed reduction in parthenogenetic development in the presence of mating partners is certainly consistent with mate-dependent reproductive plasticity. However, the current data do not clearly distinguish between:

1.	Active suppression of parthenogenesis through a signalling or recognition mechanism; and
2.	A shift in reproductive allocation toward sexual reproduction when compatible mates are available, which may me more likely?

Demonstration of transcriptional, physiological, or signalling changes associated with mate perception would considerably strengthen the claim that parthenogenesis is actively suppressed. As currently presented, I believe the authors should either moderate this conclusion or acknowledge these alternative interpretations more explicitly.

**Re:** We agree that there are alternative explanations for the observed mate-dependent reproductive plasticity. Accordingly, we have removed statements suggesting an active inhibition of parthenogenesis and allow for alternative explanations. We agree with the interpretation that a mate-recognition mechanism interacts with reproductive allocation, and we reduced speculation on the underlying mechanism (active suppression of parthenogenesis vs. enhancement of sexual reproduction).

**Ln. 21:** “Moreover, we find that the presence of potential mating partners reduces asexual development, revealing that reproductive mode is adjusted in response to reproductive opportunities.”

**Ln. 88:** “We demonstrate that the presence of the opposite sex inhibits parthenogenetic development, suggesting that a diffusible signal mediates mate-dependent reproductive plasticity between asexual and sexual reproduction.”

**Ln. 302:** “Together, these results reveal that reproductive plasticity in *L. pallida* gametophytes is mediated by mate presence, suggesting that parthenogenesis and sexual reproduction are modulated by a yet undescribed mate-recognition mechanism.”

**Ln. 335:** “This apparent paradox is resolved by our mate-exclusion experiment, which demonstrates that parthenogenesis is modulated in response to mate availability. In both sexes, parthenogenetic development increased when mating partners were absent and declined when they were present. These findings point to a mate-recognition mechanism which mediates reproductive allocation between sexual and asexual reproduction.”

**Ln. 388:** “Our experiments further indicate that parthenogenesis is regulated in response to mate availability. In both sexes, asexual development increased when mating partners were absent, suggesting that a mate-recognition mechanism mediates reproductive allocation between asexual and sexual developmental trajectories.”

3. Enough evidence from two natural populations?

The finding that parthenogenetic development was detected in two natural populations is interesting and supports the ecological relevance of the phenomenon, and more generally the authors hypotheses.

However, I am concerned that some of the wording may overstate the extent to which this pattern is maintained across the species. Specifically, the statement that the results from two populations support the presence of parthenogenesis in multiple natural populations is justified. However, this does not necessarily demonstrate that the trait is "broadly maintained" throughout the species.

The term broadly implies evidence from a larger geographic or population-level sampling effort, or alternatively, support from previous studies. Unless such evidence exists, I recommend either tempering this wording or providing additional justification from the literature. The current data support occurrence in multiple populations, but not necessarily widespread maintenance across the species as a whole.

**Re:** We agree that our previous wording could be interpreted as implying that parthenogenetic capacity is broadly maintained across the species, which cannot be established from two populations alone. We have therefore removed the term “broadly” (**ln. 21, ln. 272**) and now explicitly state that our observations concern the two natural populations sampled. However, we would like to emphasize that parthenogenetic capacity was detected in both populations and in the majority of gametophytes examined within these populations (**ln. 268**). Thus, while our data do not establish how widespread this trait is across the species’ geographic range, the consistency of the observations across two independent populations indicates that parthenogenetic capacity is not restricted to a single population or exceptional genotype and supports its ecological relevance under natural conditions. We have revised the manuscript throughout to make this distinction clear.

**Ln. 20:** “We show that asexual reproduction is associated with endoreduplication and reduced fitness, while it is retained but rarely expressed in two natural populations.”

**Ln. 81:** “By combining transcriptomic analyses of sexual and asexual developmental pathways with population-level surveys in the wild, we characterize the molecular and ecological factors that govern these developmental fate decisions, with a particular focus on male parthenogenesis.”

**Ln. 272:** “These results indicate that the potential for clonal reproduction is maintained in both sexes in the natural populations examined, even though its rare realization among adult sporophytes suggests that asexual reproduction may be ecologically or competitively disadvantaged relative to sexual reproduction.”

Reviewer #2:

This manuscript, entitled "Life-cycle plasticity enables conditional asexual reproduction in a kelp," investigates the regulation of life cycle plasticity and reproductive mode in the South African kelp Laminaria pallida. By integrating developmental experiments, transcriptomics (RNA seq and WGCNA), and population genetics (microsatellite markers), the study reports that both male and female gametes of this species are capable of parthenogenetic development, that parthenogenesis occurs readily in laboratory culture but was not detected in either of the two natural populations surveyed, and that the presence of a mating partner suppresses parthenogenetic development. 
Overall, the study employs a multi scale design from the molecular to the individual and population levels, addressing the question of how sexual reproduction is maintained in species with latent parthenogenetic capacity, and offers insights relevant to evolutionary biology, developmental biology, and ecology. 

1. The abstract states that the authors "identify the genetic networks that govern developmental state transitions"; co-expression modules and differential expression are correlative, and no functional test (knockdown, mutant, complementation) is presented, so "govern" should be replaced by wording such as "characterise the transcriptional signatures associated with".

**Re:** We thank the reviewer for the positive comments. We have changed the abstract accordingly.

**Ln. 17:** “By combining gene expression analyses with developmental experiments and natural population-level approaches, we characterize the transcriptional signatures associated with developmental state transitions in both sexual and asexual contexts.”

2. The transcriptomic analyses identify gene modules and enriched GO terms associated with parthenogenetic development, but fail to pinpoint specific key regulatory genes, limiting the depth of mechanistic insight. The TALE homeodomain transcription factors SAMSARA and OUROBOROS do not show the expected stage specific expression in L. pallida; although this negative result is intriguing, the authors' inference that L. pallida and Ectocarpus differ in their regulatory mechanisms based on this observation is insufficiently supported.

**Re:** We agree that our transcriptomic analyses do not identify a single key regulator of parthenogenetic development. Rather, they reveal broader transcriptional programs and gene co-expression modules associated with the alternative developmental trajectories, providing candidate processes and genes for future functional investigation. We have revised the text to make this distinction clearer and to avoid implying a level of mechanistic resolution that cannot be obtained from transcriptomic data alone.

We also agree that the expression patterns of *SAMSARA* and *OUROBOROS* are not sufficient to conclude that life-cycle regulation differs mechanistically between *L. pallida* and *Ectocarpus*. We found their continuous expression throughout male and female development intriguing given their established roles in *Ectocarpus*, but several explanations remain possible, including regulation at the post-transcriptional or protein level. We have therefore removed the inference of divergent regulatory mechanisms and revised the paragraph to explicitly present these alternative possibilities.

**Ln. 347:** “However, unlike in *Ectocarpus* [16,17], both *SAMSARA* and *OUROBOROS* are continuously expressed throughout male and female development in *Laminaria pallida*. Whether *SAMSARA* and *OUROBOROS* are subject to regulation at other levels (e.g., post-transcriptional regulation [42]), or whether parthenogenetic development in *L. pallida* involves additional regulatory factors, remains to be investigated.”

3. The mate‑signalling experiment lacks unidirectional compartmentalised controls (i.e., female‑only medium flowing to males, and male‑only medium flowing to females), making it impossible to determine whether the inhibitory signals secreted by females and males are the same molecule, and thus the molecular basis of bidirectional regulation remains ambiguous. Furthermore, the experimental design does not exclude confounding factors such as nutrient competition or accumulation of metabolic waste; therefore, it cannot be definitively concluded that the suppression is caused by mate‑specific signals rather than by general environmental stress.

**Re:** We agree that our experiments do not establish whether the effects mediated by males and females involve the same molecular signal. This was not the aim of the experiment, which was designed to test whether the presence of the opposite sex influences parthenogenetic development without direct physical contact. Nevertheless, we recognize that our use of the term “bidirectional” could be interpreted as implying a common signalling mechanism. We have therefore removed this term (**ln. 304**) and revised the text to distinguish the reciprocal biological response we observe from the still unknown molecular nature of the underlying signal(s).

We would, however, like to clarify that the experimental design specifically controlled for potential effects of nutrient competition and accumulation of metabolic waste. Gametophytes were sown at defined densities on both sides of the mesh in all treatments. In the mate-presence treatment, males and females were placed on opposite sides of the mesh, whereas in the mate-absence controls, both compartments contained gametophytes of the same sex (**ln. 626**). Thus, the overall density of gametophytes and associated nutrient consumption and metabolic activity were comparable between treatments; the principal experimental variable was the sexual identity of the gametophytes in the opposite compartment. In addition, 20 mL of the total 30 mL culture medium was replaced weekly with fresh medium to limit nutrient depletion and accumulation of metabolic products.

We have revised the Methods to make these controls and culture conditions more explicit and now refer directly to the relevant culture procedure in both the “Developmental time course” (**ln. 499**) and “Mate exclusion experiment” (**ln. 620**) sections.

**Ln. 626:** “To this end, we seeded five replicate dishes of each male strain Lp_M1 – Lp_M4, and the female wild type Lp_F1 on either side of the mesh as a negative control (absence of mate) using the method described above. As a mate presence treatment, we seeded further five replicate dishes of the four male strains only on one side, while the other side was seeded with the female wild type. Gametophyte filaments were sown to a target density of 500 gametophytes cm^-2^ in 30 mL 50% PES. Once per week, 20 mL of culture medium were replenished with 50% PES.”

While identification of the molecule(s) responsible will require future experiments, we believe that the controlled-density design supports our conclusion that the observed suppression is associated with the presence of the opposite sex rather than with a general effect of culture density, nutrient depletion or metabolic waste accumulation.

4. The 5 µm nylon mesh separating the compartments (L576) needs to be validated. Kelp spermatozoids are of comparable size and are flagellated; if any cross the barrier, sporophytes forming on the female side in the "mate present" treatment may be zygotic rather than parthenogenetic, which would produce exactly the reported pattern without any suppression of parthenogenesis. The sex-specific PCR markers already established in this study (L421-426) provide a direct test: sporophytes recovered from the female compartment under mate presence should carry the U marker only. Without this control the female half of the "bidirectional" conclusion cannot be evaluated.

**Re:** We appreciate the reviewer raising this point. We agree that, given the size of kelp sperm cells relative to the 5 µm mesh, the mesh itself cannot be considered definitive evidence that sperm passage is physically impossible. However, several independent observations argue strongly against fertilization being responsible for the sporophytes observed on the female side of the experiment.

First, fertilization and female parthenogenesis produce very different reproductive outcomes in our experiments. As shown in **Figure 1**, reproductive success following fertilization is substantially higher than that of female parthenogenesis. If sperm crossing the mesh were responsible for a significant proportion of sporophytes in the mate-presence treatment, we would therefore expect the presence of males to increase, rather than decrease, sporophyte production. Instead, we observe the opposite pattern: significantly more sporophytes are produced by females in the absence of males than in their presence (**Figure 5**).

Second, the effect of mate presence is already apparent at egg release, which precedes any possible fertilization event. Females released more eggs in the absence than in the presence of males. Sperm-mediated fertilization of released eggs therefore cannot account for this effect, supporting the conclusion that the presence of males influences female reproductive behavior before fertilization.

Third, the sporophytes recovered in these experiments displayed morphological characteristics associated with parthenogenetic development: they were frequently malformed and were not attached to a maternal gametophyte (**Figure R1**; Dries et al., 2024; tom Dieck, 1992). Together, these observations provide an additional, independent indication that the sporophytes scored in the experiment were of parthenogenetic rather than zygotic origin.

We agree that genotyping individual sporophytes with the sex-specific markers would provide an additional direct test of their origin. These samples are no longer available for retrospective genotyping, and we therefore cannot provide this additional analysis for the present experiment. We have instead revised the manuscript to acknowledge explicitly that passage of occasional sperm through the mesh cannot be formally excluded. Importantly, however, the direction of the observed effect, its occurrence already at the level of egg release, and the morphology of the resulting sporophytes all independently argue against sperm passage and fertilization as an explanation for the observed suppression of female parthenogenetic development.

Note that we have also removed the term “bidirectional” as described in our response to the previous comment, to avoid making conclusions beyond what can be established from the current experiment.

We have added a caveat in **line 299**:

“While we cannot fully exclude that some spermatozoids may have passed the mesh (see gamete size in [30]), the abnormal morphology of sporophytes [20,32] and their lack of attachment to the maternal oogonium [22] strongly suggested parthenogenetic origin.”

And we add in **ln. 391**:

“In the female strain, the increased parthenogenesis in absence of males contrasts the higher reproductive success of fertilisation in the developmental time course experiment.”

**Figure R1.** Morphological differences between (A) zygotic sporophytes and (B) partheno-sporophytes of *L. pallida*. Note the regular morphology and basal attachment to the empty gametophyte oogonium in (A) and the free-floating, deformed partheno-sporophyte in (B).

5. Fitness is invoked multiple times throughout the manuscript, yet the assessment remains relatively simplistic. In particular, the authors' suggestion that parthenogenesis incurs "little additional cost" lacks empirical support from measurements such as energy allocation, gamete viability duration, or other relevant physiological parameters.

**Re:** We thank the reviewer for raising this point. We agree that our original wording did not sufficiently distinguish between the fitness consequences of parthenogenetic development, which we assess experimentally, and the potential cost of maintaining parthenogenetic capacity in male gametophytes.

Our experiments provide clear evidence that parthenogenesis is associated with substantial fitness consequences at the sporophyte stage: parthenosporophytes show markedly impaired growth and development compared with sexually produced sporophytes. We therefore do not consider our conclusions regarding the reduced performance of parthenogenetic offspring to be speculative.

The reviewer is correct, however, that we did not directly quantify the energetic or physiological cost to male gametophytes of retaining the capacity for parthenogenesis. Our suggestion that this capacity may incur relatively little additional cost was based on the timing of parthenogenetic development: male gametophytes produce functional sperm (our experiments; Liboureau et al., 2024), and in our experiments most parthenosporophytes developed only after the main period of gamete release. Thus, parthenogenesis appears to provide an alternative developmental route that is predominantly expressed after opportunities for sexual reproduction have declined, rather than replacing investment in sexual reproduction.

We have revised the text to clarify this distinction and to avoid implying that the physiological cost of maintaining parthenogenetic capacity was directly measured.

**Ln. 413:** “Yet the evolutionary maintenance of this trait may not require substantial fitness benefits. Male gametophytes produce functional sperm [27], and in our experiments most parthenosporophytes developed only after the main period of gamete release. Thus, investment in asexual development occurs predominantly after opportunities for sexual reproduction have declined. Although we did not directly quantify the physiological costs of maintaining parthenogenetic capacity, this temporal separation may limit interference with sexual reproduction while retaining an alternative route to reproduction when mating fails.”

6. Given that the authors have already proposed a speculative link between warming and parthenogenesis, and that the experimental system is already established, would it be possible to conduct controlled laboratory experiments with a temperature gradient to directly test the effect of temperature on the efficiency of parthenogenetic conversion in both male and female gametophytes?

**Re:** We thank the reviewer for this interesting suggestion. While we consider this additional experiment to be beyond the scope for our manuscript, we now expand the discussion to include the temperature-dependent developmental experiments by Martins et al. (2019) and temperature trends in the Benguela upwelling system.

**Ln. 78:** “*L. pallida* occurs subtidally along the Atlantic coast of South Africa and Namibia, where the Benguela current fuels coastal upwelling of cold, nutrient-rich water [26].”

**Ln. 398:** “Evidence for such a role in natural populations, however, remains limited. We detected a weak but significant deviation from random mating in the Namibian population that could reflect cryptic clonality. Whether this pattern is related to local environmental conditions remains unclear. In summer, sea surface temperatures at Swakopmund reach 20°C and above (**Figure S2E;** see also [55]), a threshold that likely impairs growth and reproduction in *L. pallida* [32]. While sporophytes may reproduce year-round [57], gametophyte reproduction is optimal at 12°C [32], likely facilitating juvenile sporophyte recruitment in cold seasons [57] or upwelling periods. Despite warming of the northern Benguela region [58], however, the species’ northernmost population at Rocky Point, Namibia, has persisted since it was first documented in 1970 [59,60]. Whether the relative importance of asexual developmental pathways increases towards the distributional range limit warrants further investigation.”

7. Three microsatellite loci show evidence of null alleles, and the authors choose to analyse the data without correction, arguing that the estimated frequencies are low and inconsistent across populations. While this approach may be acceptable, the potential impact of null alleles on the results should be more thoroughly discussed.

**Re:** We thank the reviewer for this suggestion. We agree that null alleles can lead to an underestimation of heterozygosity and potentially inflate estimates of inbreeding and genetic differentiation. We have therefore added an explicit caveat to the Methods. In our dataset, estimated null allele frequencies were low (<8%) and, importantly, were not consistently associated with the same loci across populations. In addition, as pointed out by Chapuis and Estoup (2007) “the presence of null alleles may be particularly problematic in studies comparing different sets of populations with different frequencies of null alleles and/or patterns of gene flow, especially when one or several population sets are characterized by low levels of gene flow.” As our study compares only two populations, we therefore considered it unlikely that they would systematically bias our population-level comparisons. Finally, Putman and Carbone (2014) noted that “although frequencies of null alleles up to 8% may cause only minimal bias in estimation of some population genetic parameters, correction may not sufficiently reduce bias for inferring population structure and may actually exacerbate it”.

We therefore chose to retain these loci because excluding three markers would substantially reduce the information available from an already limited microsatellite dataset. Nevertheless, we now explicitly acknowledge the potential effect of null alleles and interpret the population-genetic estimates accordingly.

**Ln. 585:** “However, estimated null allele frequencies were low (< 8%; [94]) and were not consistent across populations. We therefore retained these loci, as excluding three markers would substantially reduce the information content of the dataset. We acknowledge that null alleles can lead to underestimation of heterozygosity and an inflation of estimates of genetic differentiation; however, given their low and inconsistent frequencies, and considering that only two populations were compared, we expect their potential bias to have a limited effect on the population-level patterns examined here.”

8. The manuscript only briefly refers to the preliminary morphological observations of male parthenogenesis in L. pallida reported by Liboureau et al. (2024). It would be beneficial to explicitly contrast the scope, experimental dimensions, and incremental novelty of the present study with that earlier work, either in the Introduction or in the Discussion.

**Re:** We thank the author for this suggestion and have now highlighted the contribution of Liboureau et al. (2024) as the first characterisation of male parthenogenesis in *L. pallida* in the introduction and discussion.

**Ln. 79:** “Gametes of both sexes of *L. pallida* have recently been shown to initiate sporophyte development without fertilisation through parthenogenesis [27]. By combining transcriptomic analyses of sexual and asexual developmental pathways with population-level surveys in the wild, we characterize the molecular and ecological factors that govern these developmental fate decisions, with a particular focus on male parthenogenesis.”

**Ln. 315:** “While male parthenogenesis had been characterized in sibling gametophytes derived from one *L. pallida* sporophyte [27], our gametophytes collected from more than 25 sporophytes revealed that almost all lines tested were capable of parthenogenetic development under laboratory conditions.”

9. No assembly or annotation statistics are reported. Assembly size, contig/scaffold N50, BUSCO completeness (stramenopiles_odb10), number of predicted gene models and repeat content should all be given, since every downstream expression result rests on this annotation; the choice of the "land_plant" lineage model in Helixer for a brown alga (L452) should likewise be justified or benchmarked. Moreover, the long-read assembly was generated from two female individuals (L435-436).

**Re:** We appreciate the request to include genome assembly and annotation statistics and have included the information in **Supplementary Table S7**. The “land_plant” lineage model in Helixer was chosen because it represents the nearest lineage to brown algae out of the available trained models: invertebrates, vertebrates, fungi, land plants (<https://github.com/usadellab/Helixer#goal>). Training a model for brown algae would be beyond the scope of this manuscript. In addition, we added the information that any mRNA models without RNAseq evidence were removed. The median RNAseq mapping rate was 98.98%, which we now also report in Supplementary Table S7.

**Ln. 492:** “Structural gene annotation was performed by Helixer v0.3.6 [78] (--linage land_plant as the trained model most closely related to brown algae) and funannotate. AGAT agat_sp_merge_annotations.pl v1.5.1 [79] was used to merge the gene models of Helixer and funannotate. mRNA models spanning over >3 different gene models and models without RNAseq evidence were removed. TE filtered protein sequences of each mRNA model were extracted and analysed by InterProScan [80], mapped by Diamond [81] to the NCBI blast nr database, Araport11 [82] and the *Ectocarpus* sp7 annotation. Genome assembly and annotation statistics are collated in **Supplementary Table S7**.”

The long-read assembly was generated from two female individuals due to more effective HMW DNA isolation from female compared to male gametophytes. Nevertheless, a male gametophyte genome was assembled from short reads and scaffolded with the long-read assembly (**lns. 480, 488**) to yield a hybrid assembly.

Reviewer #3:

The manuscript by Liesner et al. characterizes life cycle transitions in the kelp Laminaria pallida.  The authors describe a form of latent parthenogenesis which they show is genetically regulated and a possible form of reproductive assurance. The authors study gene expression and development in isolated gametophyte cultures which allows them to estimate the male and female genetic pathways as well as the manner and timing of parthenogenesis. Apparently, the parthenogenetic sporophytes are not as fit as sexually produced ones. Overall, I found the study comprehensive and convincing as a characterization of a species sexual developmental processes. The paper is written clearly and with well explained arguments. I only found a handful of minor criticisms that the authors should consider.

1. The flow cytometry data are a bit messy, but after looking at it a bit more, I see that in general it supports the authors claims.  I think the authors should explain either in the text or legend of this supplementary figure how they are interpreting the data, why there are two peaks in some but not all samples, and explain a bit about the variation in location of peaks.

**Re:** We thank the reviewer for the suggestion to include more information in the figure legend.

**Ln. 1008 / Supplementary Figure S1:** “Peaks between DNA-H of 1.0–1.5 were interpreted as 1C (see presumably haploid gametophyte samples GA), peaks from 1.8–2.7 as 2C (multiplication factor 1.8). Peaks may vary in position due to variation between independent measurements. We interpret multiple peaks as mitosis in actively dividing cells.”

2. The gene coexpression network analysis is great.  However, the GO term enrichment is not very well developed as there are categories of enriched function in different sets, but these are not interpreted. There are 4 supplementary figures of GO term enrichment, and the authors don’t really explain the biology of what they find.  How can we interpret these results?  Are there hypotheses to explain these enrichments?  For example, this is not expanded on in the discussion section.

**Re:** We thank the reviewer for this helpful suggestion. We agree that the biological context of the GO term enrichment analyses was insufficiently developed in the original manuscript. We have therefore expanded the Discussion to interpret the main enrichment patterns in the context of brown algal development and the developmental differences observed in our study.

At the same time, we have deliberately remained cautious in interpreting individual GO categories. Functional annotation remains incomplete in brown algae, and many GO assignments are based on sequence similarity rather than direct functional characterization. We therefore prefer not to infer specific biological mechanisms from individual enriched terms in the absence of experimental evidence. Instead, we now highlight those broader patterns for which there is a plausible biological or developmental context, while making clear where the interpretation remains hypothetical.

**Ln. 356:** “The enrichment of GO terms related to reactive oxygen species (ROS), which play important roles in embryogenesis in both plants and brown algae [45,46], suggests a potential involvement of ROS-related signalling in (partheno-)sporophyte development. Enrichment of photosynthesis- and metabolism-related GO terms may reflect changes in energy metabolism associated with the morphological transition from filamentous to sheet-like growth, the latter potentially enabling more efficient photosynthesis [47].”

**Ln. 366:** “GO terms enriched in association with male parthenogenesis further point to a potential role for post-transcriptional regulation, which has recently been shown to contribute to the fine-tuning of life-cycle stage identity in *Ectocarpus* [42].”

3. Lines 59-60. The authors say, " Whether these parthenosporophytes consistently execute the diploid sporophyte developmental program, however, remains unknown." I think at this point in the paper, it would be useful to explain what is uncertain in more specific terms as it is unclear what might not be executed.

**Re:** We agree that the previous sentence did not make sufficiently clear what remained unknown. Our intention was to distinguish the acquisition of sporophyte identity, which is well established in haploid parthenosporophytes, from the extent to which these individuals subsequently recapitulate the developmental program of diploid, zygote-derived sporophytes. Previous developmental studies in *Ectocarpus* indicate that parthenosporophytes acquire sporophyte identity but do not entirely recapitulate the development of diploid, zygote-derived sporophytes (Bothwell et al., 2010). However, whether and to what extent these alternative developmental trajectories differ at the transcriptomic level has not been systematically investigated. We have therefore revised this paragraph to clarify that our study asks to what extent the transcriptional and developmental programs associated with parthenogenesis resemble, or diverge from, those of sexual embryogenesis.

**Ln. 56:** “In *Ectocarpus*, the sporophyte developmental program is activated in both zygotes and unfused gametes through the dimerization of two TALE homeodomain transcription factors [16,17], uncoupling life-cycle identity from ploidy. As a result, both male and female gametes can develop parthenogenetically into haploid parthenosporophytes. Although these individuals acquire sporophyte identity, previous observations suggest that parthenosporophyte development does not fully recapitulate that of diploid, zygote-derived sporophytes [11,18]. However, the extent to which the developmental and transcriptional programs initiated during parthenogenesis resemble those of sexual embryogenesis, and where they diverge, has not been systematically investigated.”

4. Lines 155-157. The authors say "Despite three independent induction attempts, parthenosporophytes remained infertile under laboratory conditions." Earlier the authors say that the parthenosporophytes show "substantially reduced fitness", but infertile to me implies zero fitness.

**Re:** We thank the reviewer for pointing out this ambiguity. Our use of “substantially reduced fitness” referred to the pronounced growth and developmental defects observed in parthenosporophytes, which were significantly smaller and showed stunted and abnormal morphology compared with zygote-derived sporophytes. We agree, however, that the subsequent statement concerning infertility could be interpreted as implying complete loss of reproductive fitness. Our experiments were not designed to quantify reproductive fitness, and failure to induce fertility under our laboratory conditions does not demonstrate that parthenosporophytes are intrinsically sterile. We have therefore revised the text to distinguish these observations and avoid overinterpreting the failure to observe reproduction. Moreover, we replaced “substantially reduced fitness” with “substantially reduced growth and developmental performance”.

**Ln. 166:** “Despite three independent induction attempts, we did not observe the formation of reproductive structures in parthenosporophytes under our laboratory conditions.

Together, these results indicate that parthenosporophytes may arise from both male and female gametes in kelp, that their development involves endoreduplication, and that they exhibit substantially reduced growth and developmental performance compared to zygotic sporophytes formed via sexual reproduction.”

5. Line 433, mentions Figure S1C, which doesn't exist.

**Re:** Thanks for pointing this out, the results are now shown in **Figure 2C**. As the results of the sex marker PCR are mentioned in the main text (**ln. 163**), we have deleted this sentence from the Methods section.

Reviewer #4:

PBIOLOGY-D-26-01891R1
Life-cycle plasticity enables conditional asexual reproduction in a kelp

I find this manuscript to be novel, technically strong, and an important contribution to our understanding of reproductive strategies in brown seaweed. The study combines experimental manipulation, transcriptomics, and population genetics to provide a comprehensive dataset addressing a biologically relevant question. I also consider the finding that the presence of the opposite sex can reduce the frequency of parthenogenesis to be particularly novel and compelling. Overall, this is a well-written manuscript that I believe is suitable for publication in PLOS Biology. I have a few comments that I believe would further strengthen the manuscript. 

- The gametophyte strains used in this study were collected in 1993, 1999, and 2022, and have since been maintained in a germplasm collection. Could the authors discuss the potential effects that the long storage period may have had on their results? Given that some of these strains have been maintained for more than 2 decades, epigenetic changes or other long-term culture effect may have occurred and could potentially influence the results.

**Re:** We thank the reviewer for raising this point. We agree that long-term maintenance of clonal gametophyte cultures can potentially lead to genetic or non-genetic changes, and we have added a caveat to the Methods acknowledging this possibility. However, several observations argue that the main biological patterns reported here are unlikely to result from long-term culture maintenance.

First, all strains have been maintained under standardized common-garden conditions, which minimizes persistent environmental effects associated with their populations of origin. Second, and importantly, we also detected a strong capacity for parthenogenetic development in freshly collected material from natural populations (**Supplementary Table S5**; Liboureau et al., 2024). Parthenogenetic capacity is therefore clearly not restricted to long-maintained laboratory strains. Finally, the cultures used here retained normal sexual functionality, with gametes capable of successful fertilization and development, providing no indication of a general deterioration of reproductive function during culture maintenance.

We nevertheless agree that somatic mutations or persistent non-genetic changes accumulated during long-term vegetative propagation cannot be completely excluded. We have therefore added this limitation to the Methods, while clarifying that the evidence from freshly collected material strongly argues against long-term culture being responsible for the occurrence of parthenogenesis itself.

**Ln. 435:** “These conditions allow decade-long maintenance of vegetatively proliferating gametophyte cultures [63], while standardized common-garden conditions minimize environmental carry-over effects associated with the populations of origin [66,67]. Long-term vegetative maintenance may nevertheless allow the accumulation of somatic mutations or persistent non-genetic changes [68]. However, the cultures used here retained sexual functionality, and the strong parthenogenetic capacity independently observed in freshly collected natural material (Supplementary Table S5; [27]) indicates that parthenogenesis itself is not a consequence of long-term laboratory culture.”

- One aspect of the experimental design that caught my attention was the inclusion of only a single female gametophyte. Could the authors explain the rationale behind this decision?

**Re:** We acknowledge that the rationale behind our experimental design was not sufficiently clear. Female parthenogenesis has been documented in kelps and other oogamous brown algae for decades. *Laminaria pallida* is particularly interesting because a recent study showed that a high proportion of male gametophytes are also capable of parthenogenetic development (Liboureau et al., 2024). Our study was therefore specifically designed to characterize the much less understood phenomenon of **male parthenogenesis**, rather than to compare variation in parthenogenetic capacity between males and females. We included one female strain to provide a reference for female parthenogenetic development and, importantly, to generate sexual crosses with the four male strains. We have revised the Introduction and Results to make this rationale and the focus on male parthenogenesis explicit.

**Ln. 81:** “By combining transcriptomic analyses of sexual and asexual developmental pathways with population-level surveys in the wild, we characterize the molecular and ecological factors that govern these developmental fate decisions, with a particular focus on male parthenogenesis.”

**Ln. 100:** “Parthenogenetic development of female gametes has been described in many kelp species [14], whereas the capacity of male gametes to initiate sporophyte development remains poorly characterized [23,27–29]. We therefore designed our experiment primarily to characterize male parthenogenesis, using clonal cultures of four male gametophytes and one female gametophyte as a reference and for sexual crosses. To compare sexual and asexual developmental trajectories, we monitored sporophyte formation in mixed and unmixed male and female gametophyte cultures (**Table S1; Figure 1A,B**).”

- Regarding the crossing experiments, lines 462-464 indicate that only the cross M1xF1 was performed. However, the results section gives the impression that the remaining male strains were also crossed with F1. Please clarify this point in the Materials and Methods. This information is important to confirm whether all male strains are capable of successful sexual reproduction in addition to producing parthenosporophytes.

**Re:** We thank the reviewer for noticing this inconsistency in the text. We included F1xM1 as a treatment to follow fertilisation and zygotic sporophyte development in the developmental time course experiment and transcriptomic analysis (**Figures 1, 3**). In addition, we performed crosses of F1 with all male gametophyte lines in a separate experiment to characterize the viability and growth of sporophytes and to confirm sexual reproduction in all lines (**Figure 2**). This is now explained more clearly in the text.

**Ln. 154:** “A subset of male and female parthenosporophytes, as well as zygotic sporophytes derived from crosses of the female with each male gametophyte line, were isolated and cultivated for further development (see Methods).”

**Ln. 448:** “To confirm parthenogenetic and sexual reproduction in all gametophyte lines and to characterize the development of the resulting (partheno-)sporophytes, all lines were transferred to reproductive conditions in isolated and mixed (each male with Lp_F1) treatments.”

- Please replace "phot" with "photons" in lines 407 and 408.

**Ln. 445:** Done.

__________________________________________________
In compliance with data protection regulations, you may request that we remove your personal registration details at any time.  (Use the following URL: https://www.editorialmanager.com/pbiology/login.asp?a=r). Please contact the publication office if you have any questions.

---------
