# Supplementary material for: Life-cycle plasticity enables conditional asexual reproduction in a kelp
Source: PLoS Biol. 2026 Nov 12;24(11):e3004065. doi: 10.1371/journal.pbio.3004065 (PMC13759113; doi:10.1371/journal.pbio.3004065)
Supplement: Peer review history

# Editor report: Decision Letter 0

**DOI:** 10.1371/journal.pbio.3004065.r001

**Authors:** Roland Roberts

30 Jun 2026

Dear Susana,

Thank you for submitting your manuscript entitled "Life-cycle plasticity enables conditional asexual reproduction in a kelp" for consideration as a Research Article by PLOS Biology.

Your manuscript has now been evaluated by the PLOS Biology editorial staff, as well as by an academic editor with relevant expertise, and I'm writing to let you know that we would like to send your submission out for external peer review.

However, before we can send your manuscript to reviewers, we need you to complete your submission by providing the metadata that is required for full assessment. To this end, please login to Editorial Manager where you will find the paper in the 'Submissions Needing Revisions' folder on your homepage. Please click 'Revise Submission' from the Action Links and complete all additional questions in the submission questionnaire.

Once your full submission is complete, your paper will undergo a series of checks in preparation for peer review. After your manuscript has passed the checks it will be sent out for review. To provide the metadata for your submission, please Login to Editorial Manager (https://www.editorialmanager.com/pbiology) within two working days, i.e. by Jul 02 2026 11:59PM.

If your manuscript has been previously peer-reviewed at another journal, PLOS Biology is willing to work with those reviews in order to avoid re-starting the process. Submission of the previous reviews is entirely optional and our ability to use them effectively will depend on the willingness of the previous journal to confirm the content of the reports and share the reviewer identities. Please note that we reserve the right to invite additional reviewers if we consider that additional/independent reviewers are needed, although we aim to avoid this as far as possible. In our experience, working with previous reviews does save time.

If you would like us to consider previous reviewer reports, please edit your cover letter to let us know and include the name of the journal where the work was previously considered and the manuscript ID it was given. In addition, please upload a response to the reviews as a 'Prior Peer Review' file type, which should include the reports in full and a point-by-point reply detailing how you have or plan to address the reviewers' concerns.

During the process of completing your manuscript submission, you will be invited to opt-in to posting your pre-review manuscript as a bioRxiv preprint. Visit http://journals.plos.org/plosbiology/s/preprints for full details. If you consent to posting your current manuscript as a preprint, please upload a single Preprint PDF.

Feel free to email us at plosbiology@plos.org if you have any queries relating to your submission.

Kind regards,

Roli

Roland Roberts, PhD

Senior Editor

PLOS Biology

rroberts@plos.org

# Peer review: Decision Letter 1

**DOI:** 10.1371/journal.pbio.3004065.r002

**Authors:** Roland Roberts

7 Aug 2026

Dear Susana,

Thank you for your patience while your manuscript "Life-cycle plasticity enables conditional asexual reproduction in a kelp" was peer-reviewed at PLOS Biology. It has now been evaluated by the PLOS Biology editors, an Academic Editor with relevant expertise, and by four independent reviewers.

You'll see that reviewer #1 is impressed by the study, but raises a number of issues. Specifically, s/he identifies two points on which they think the evidence is insufficient; one is the case for bidirectional mate-recognition (whether this is active or passive), and one is the extrapolation from just two natural populations. Reviewer #2 also raises a number of issues where they believe that you can’t rule out alternative explanations, and more experimental data may be needed (his/her points 2, 3 and 4, regarding interspecific regulatory differences and bidirectional mate signalling). Reviewers #3 and #4 are both very positive, and only have textual requests.

In light of the reviews, which you will find at the end of this email, we would like to invite you to revise the work to thoroughly address the reviewers' reports.

Given the extent of revision needed, we cannot make a decision about publication until we have seen the revised manuscript and your response to the reviewers' comments. Your revised manuscript is likely to be sent for further evaluation by all or a subset of the reviewers.

In addition to these revisions, you will need to complete some formatting changes, which you will receive in a follow up email. A member of our team will be in touch with a set of requests shortly.

We expect to receive your revised manuscript within 3 months. Please email us (plosbiology@plos.org) if you have any questions or concerns, or would like to request an extension.

At this stage, your manuscript remains formally under active consideration at our journal; please notify us by email if you do not intend to submit a revision so that we may withdraw it.

**IMPORTANT - SUBMITTING YOUR REVISION**

Your revisions should address the specific points made by each reviewer. Please submit the following files along with your revised manuscript:

1. A 'Response to Reviewers' file - this should detail your responses to the editorial requests, present a point-by-point response to all of the reviewers' comments, and indicate the changes made to the manuscript.

*NOTE: In your point-by-point response to the reviewers, please provide the full context of each review. Do not selectively quote paragraphs or sentences to reply to. The entire set of reviewer comments should be present in full and each specific point should be responded to individually, point by point.

You should also cite any additional relevant literature that has been published since the original submission and mention any additional citations in your response.

2. In addition to a clean copy of the manuscript, please also upload a 'track-changes' version of your manuscript that specifies the edits made. This should be uploaded as a "Revised Article with Changes Highlighted" file type.

*Re-submission Checklist*

When you are ready to resubmit your revised manuscript, please refer to this re-submission checklist: https://plos.io/Biology_Checklist

To submit a revised version of your manuscript, please go to https://www.editorialmanager.com/pbiology/ and log in as an Author. Click the link labelled 'Submissions Needing Revision' where you will find your submission record.

Please make sure to read the following important policies and guidelines while preparing your revision:

*Published Peer Review*

Please note while forming your response, if your article is accepted, you may have the opportunity to make the peer review history publicly available. The record will include editor decision letters (with reviews) and your responses to reviewer comments. If eligible, we will contact you to opt in or out. Please see here for more details:

https://blogs.plos.org/plos/2019/05/plos-journals-now-open-for-published-peer-review/

*PLOS Data Policy*

Please note that as a condition of publication PLOS' data policy (http://journals.plos.org/plosbiology/s/data-availability) requires that you make available all data used to draw the conclusions arrived at in your manuscript. If you have not already done so, you must include any data used in your manuscript either in appropriate repositories, within the body of the manuscript, or as supporting information (N.B. this includes any numerical values that were used to generate graphs, histograms etc.). For an example see here: http://www.plosbiology.org/article/info%3Adoi%2F10.1371%2Fjournal.pbio.1001908#s5

*Blot and Gel Data Policy*

We require the original, uncropped and minimally adjusted images supporting all blot and gel results reported in an article's figures or Supporting Information files. We will require these files before a manuscript can be accepted so please prepare them now, if you have not already uploaded them. Please carefully read our guidelines for how to prepare and upload this data: https://journals.plos.org/plosbiology/s/figures#loc-blot-and-gel-reporting-requirements

*Protocols deposition*

To enhance the reproducibility of your results, we recommend that if applicable you deposit your laboratory protocols in protocols.io, where a protocol can be assigned its own identifier (DOI) such that it can be cited independently in the future. Additionally, PLOS ONE offers an option for publishing peer-reviewed Lab Protocol articles, which describe protocols hosted on protocols.io. Read more information on sharing protocols at https://plos.org/protocols?utm_medium=editorial-email&utm_source=authorletters&utm_campaign=protocols

Thank you again for your submission to our journal. We hope that our editorial process has been constructive thus far, and we welcome your feedback at any time. Please don't hesitate to contact us if you have any questions or comments.

Sincerely,

Roli

Roland Roberts, PhD

Senior Editor

PLOS Biology

rroberts@plos.org

------------------------------------

REVIEWERS' COMMENTS:

Reviewer #1:

The paper by Liesner and colleagues presents a thorough investigation of parthenogenesis versus sexual reproduction in a species of brown alga.

Overall, I have no major concerns regarding the methodologies employed or the associated statistical analyses. The study is well written, clear, and relatively accessible even to readers who are not specialists in algal biology. Several of the results are both exciting and compelling, others required additional support (in my opinion).

My main concerns relate to the strength of some of the conclusions and whether they should be toned down or revised to better reflect the available evidence. A second concern is whether findings from a single algal species can reasonably be interpreted as representing a broader or universal pattern across lineages exhibiting both parthenogenetic and sexual reproductive modes.

Specific comments are provided below.

1. Novelty and broader significance:

One of the strongest and most novel findings of this study is the demonstration that male as well as female gametes possess autonomous sporophyte developmental capacity. Female gamete parthenogenesis has already been reported in several brown algal systems; therefore, the demonstration of male gamete-derived sporophyte development, together with the identification of endoreduplication as a mechanism underlying this developmental transition, represents an important advance.

However, the authors should be cautious when emphasizing the broader novelty of the finding that parthenogenetic sporophytes exhibit fitness disadvantages relative to sexually derived diploid sporophytes. Similar fitness costs associated with parthenogenesis have been documented in a variety of other taxa. Consequently, the principal novelty of the study lies not in demonstrating fitness disadvantages per se, but rather in revealing these patterns within this algal system and linking them to the underlying developmental and genomic mechanisms.

2. Developmental reprogramming and mate-recognition mechanisms:

The observation that male gamete-derived developmental programs become transcriptionally "less male" and shift toward a female-like or zygotic state provides a compelling mechanistic insight into developmental reprogramming. This finding has potentially broad implications for our understanding of developmental plasticity, sex-specific gene regulation, and the evolution of asexual reproduction.

However, I am less convinced by the strength of the evidence supporting the conclusion that there is "a bidirectional mate-recognition mechanism that promotes sexual reproduction while actively suppressing parthenogenesis."

The observed reduction in parthenogenetic development in the presence of mating partners is certainly consistent with mate-dependent reproductive plasticity. However, the current data do not clearly distinguish between:

1.Active suppression of parthenogenesis through a signalling or recognition mechanism; and

2.A shift in reproductive allocation toward sexual reproduction when compatible mates are available, which may me more likely?

Demonstration of transcriptional, physiological, or signalling changes associated with mate perception would considerably strengthen the claim that parthenogenesis is actively suppressed. As currently presented, I believe the authors should either moderate this conclusion or acknowledge these alternative interpretations more explicitly.

3. Enough evidence from two natural populations?

The finding that parthenogenetic development was detected in two natural populations is interesting and supports the ecological relevance of the phenomenon, and more generally the authors hypotheses.

However, I am concerned that some of the wording may overstate the extent to which this pattern is maintained across the species. Specifically, the statement that the results from two populations support the presence of parthenogenesis in multiple natural populations is justified. However, this does not necessarily demonstrate that the trait is "broadly maintained" throughout the species.

The term broadly implies evidence from a larger geographic or population-level sampling effort, or alternatively, support from previous studies. Unless such evidence exists, I recommend either tempering this wording or providing additional justification from the literature. The current data support occurrence in multiple populations, but not necessarily widespread maintenance across the species as a whole.

Reviewer #2:

This manuscript, entitled "Life-cycle plasticity enables conditional asexual reproduction in a kelp," investigates the regulation of life cycle plasticity and reproductive mode in the South African kelp Laminaria pallida. By integrating developmental experiments, transcriptomics (RNA seq and WGCNA), and population genetics (microsatellite markers), the study reports that both male and female gametes of this species are capable of parthenogenetic development, that parthenogenesis occurs readily in laboratory culture but was not detected in either of the two natural populations surveyed, and that the presence of a mating partner suppresses parthenogenetic development.

Overall, the study employs a multi scale design from the molecular to the individual and population levels, addressing the question of how sexual reproduction is maintained in species with latent parthenogenetic capacity, and offers insights relevant to evolutionary biology, developmental biology, and ecology.

1. The abstract states that the authors "identify the genetic networks that govern developmental state transitions"; co-expression modules and differential expression are correlative, and no functional test (knockdown, mutant, complementation) is presented, so "govern" should be replaced by wording such as "characterise the transcriptional signatures associated with".

2. The transcriptomic analyses identify gene modules and enriched GO terms associated with parthenogenetic development, but fail to pinpoint specific key regulatory genes, limiting the depth of mechanistic insight. The TALE homeodomain transcription factors SAMSARA and OUROBOROS do not show the expected stage specific expression in L. pallida; although this negative result is intriguing, the authors' inference that L. pallida and Ectocarpus differ in their regulatory mechanisms based on this observation is insufficiently supported.

3. The mate‑signalling experiment lacks unidirectional compartmentalised controls (i.e., female‑only medium flowing to males, and male‑only medium flowing to females), making it impossible to determine whether the inhibitory signals secreted by females and males are the same molecule, and thus the molecular basis of bidirectional regulation remains ambiguous. Furthermore, the experimental design does not exclude confounding factors such as nutrient competition or accumulation of metabolic waste; therefore, it cannot be definitively concluded that the suppression is caused by mate‑specific signals rather than by general environmental stress.

4. The 5 µm nylon mesh separating the compartments (L576) needs to be validated. Kelp spermatozoids are of comparable size and are flagellated; if any cross the barrier, sporophytes forming on the female side in the "mate present" treatment may be zygotic rather than parthenogenetic, which would produce exactly the reported pattern without any suppression of parthenogenesis. The sex-specific PCR markers already established in this study (L421-426) provide a direct test: sporophytes recovered from the female compartment under mate presence should carry the U marker only. Without this control the female half of the "bidirectional" conclusion cannot be evaluated.

5. Fitness is invoked multiple times throughout the manuscript, yet the assessment remains relatively simplistic. In particular, the authors' suggestion that parthenogenesis incurs "little additional cost" lacks empirical support from measurements such as energy allocation, gamete viability duration, or other relevant physiological parameters.

6. Given that the authors have already proposed a speculative link between warming and parthenogenesis, and that the experimental system is already established, would it be possible to conduct controlled laboratory experiments with a temperature gradient to directly test the effect of temperature on the efficiency of parthenogenetic conversion in both male and female gametophytes?

7. Three microsatellite loci show evidence of null alleles, and the authors choose to analyse the data without correction, arguing that the estimated frequencies are low and inconsistent across populations. While this approach may be acceptable, the potential impact of null alleles on the results should be more thoroughly discussed.

8. The manuscript only briefly refers to the preliminary morphological observations of male parthenogenesis in L. pallida reported by Liboureau et al. (2024). It would be beneficial to explicitly contrast the scope, experimental dimensions, and incremental novelty of the present study with that earlier work, either in the Introduction or in the Discussion.

9. No assembly or annotation statistics are reported. Assembly size, contig/scaffold N50, BUSCO completeness (stramenopiles_odb10), number of predicted gene models and repeat content should all be given, since every downstream expression result rests on this annotation; the choice of the "land_plant" lineage model in Helixer for a brown alga (L452) should likewise be justified or benchmarked. Moreover, the long-read assembly was generated from two female individuals (L435-436).

Reviewer #3:

The manuscript by Liesner et al. characterizes life cycle transitions in the kelp Laminaria pallida. The authors describe a form of latent parthenogenesis which they show is genetically regulated and a possible form of reproductive assurance. The authors study gene expression and development in isolated gametophyte cultures which allows them to estimate the male and female genetic pathways as well as the manner and timing of parthenogenesis. Apparently, the parthenogenetic sporophytes are not as fit as sexually produced ones. Overall, I found the study comprehensive and convincing as a characterization of a species sexual developmental processes. The paper is written clearly and with well explained arguments. I only found a handful of minor criticisms that the authors should consider.

1. The flow cytometry data are a bit messy, but after looking at it a bit more, I see that in general it supports the authors claims. I think the authors should explain either in the text or legend of this supplementary figure how they are interpreting the data, why there are two peaks in some but not all samples, and explain a bit about the variation in location of peaks.

2. The gene coexpression network analysis is great. However, the GO term enrichment is not very well developed as there are categories of enriched function in different sets, but these are not interpreted. There are 4 supplementary figures of GO term enrichment, and the authors don't really explain the biology of what they find. How can we interpret these results? Are there hypotheses to explain these enrichments? For example, this is not expanded on in the discussion section.

3. Lines 59-60. The authors say, " Whether these parthenosporophytes consistently execute the diploid sporophyte developmental program, however, remains unknown." I think at this point in the paper, it would be useful to explain what is uncertain in more specific terms as it is unclear what might not be executed.

4. Lines 155-157. The authors say "Despite three independent induction attempts, parthenosporophytes remained infertile under laboratory conditions." Earlier the authors say that the parthenosporophytes show "substantially reduced fitness", but infertile to me implies zero fitness.

5. Line 433, mentions Figure S1C, which doesn't exist.

Reviewer #4:

PBIOLOGY-D-26-01891R1

Life-cycle plasticity enables conditional asexual reproduction in a kelp

I find this manuscript to be novel, technically strong, and an important contribution to our understanding of reproductive strategies in brown seaweed. The study combines experimental manipulation, transcriptomics, and population genetics to provide a comprehensive dataset addressing a biologically relevant question. I also consider the finding that the presence of the opposite sex can reduce the frequency of parthenogenesis to be particularly novel and compelling. Overall, this is a well-written manuscript that I believe is suitable for publication in PLOS Biology. I have a few comments that I believe would further strengthen the manuscript.

- The gametophyte strains used in this study were collected in 1993, 1999, and 2022, and have since been maintained in a germplasm collection. Could the authors discuss the potential effects that the long storage period may have had on their results? Given that some of these strains have been maintained for more than 2 decades, epigenetic changes or other long-term culture effect may have occurred and could potentially influence the results.

- One aspect of the experimental design that caught my attention was the inclusion of only a single female gametophyte. Could the authors explain the rationale behind this decision?

- Regarding the crossing experiments, lines 462-464 indicate that only the cross M1xF1 was performed. However, the results section gives the impression that the remaining male strains were also crossed with F1. Please clarify this point in the Materials and Methods. This information is important to confirm whether all male strains are capable of successful sexual reproduction in addition to producing parthenosporophytes.

- Please replace "phot" with "photons" in lines 407 and 408.

# Author comment: Author response to Decision Letter 2

**DOI:** 10.1371/journal.pbio.3004065.r003

25 Aug 2026

Attachment Submitted filename: 20260824_response_to_reviewers_edits.docx

# Peer review: Decision Letter 2

**DOI:** 10.1371/journal.pbio.3004065.r004

**Authors:** Roland Roberts

29 Sep 2026

Dear Susana,

Thank you for your patience while we considered your revised manuscript "Life-cycle plasticity enables conditional asexual reproduction in a kelp" for publication as a Research Article at PLOS Biology. This revised version of your manuscript has been evaluated by the PLOS Biology editors, the Academic Editor and two of the original reviewers.

Based on the reviews, we are likely to accept this manuscript for publication, provided you satisfactorily address the following data and other policy-related requests.

IMPORTANT - please attend to the following:

a) Please make the data deposited at NCBI and EDMOND publicly available before the manuscript enters Production.

b) Please address my Data Policy requests below; specifically, we need you to supply the numerical values underlying Figs 1E, 2B, 3AB, 4BC, 5AB, S1, S2CDE, S3, S4AB, S5AB, S6ABC, S7ABC,S8ABC, S9ABC, either as a supplementary data file or as a permanent DOI’d deposition.

c) Please clearly cite the location of the data in all relevant main and supplementary figure legends. For example, you can add: “The data underlying this figure can be found at doi.org/10.xxx/zenodo.xxxxx.” We understand that this may appear repetitive, but it helps make the figures and their legends more self-contained.

d) Please include the URLs of your funders in the Financial Disclosure statement.

e) Please make any custom code available either as part of your data deposition or as a supplementary file.

f) Please make sure to provide the uncropped gels (Fig. 2C) as supplementary files.

As you address these items, please take this last chance to review your reference list to ensure that it is complete and correct. If you have cited papers that have been retracted, please include the rationale for doing so in the manuscript text, or remove these references and replace them with relevant current references. Any changes to the reference list should be mentioned in the cover letter that accompanies your revised manuscript.

In addition to these revisions, you may need to complete some formatting changes, which you will receive in a follow up email. A member of our team will be in touch with a set of requests shortly. If you do not receive a separate email within a few days, please assume that checks have been completed, and no additional changes are required.

We expect to receive your revised manuscript within two weeks.

To submit your revision, please go to https://www.editorialmanager.com/pbiology/ and log in as an Author. Click the link labelled 'Submissions Needing Revision' to find your submission record. Your revised submission must include the following:

- a cover letter that should detail your responses to any editorial requests, if applicable, and whether changes have been made to the reference list

- a Response to Reviewers file that provides a detailed response to the reviewers' comments (if applicable, if not applicable please do not delete your existing 'Response to Reviewers' file.)

- a track-changes file indicating any changes that you have made to the manuscript.

NOTE: If Supporting Information files are included with your article, note that these are not copyedited and will be published as they are submitted. Please ensure that these files are legible and of high quality (at least 300 dpi) in an easily accessible file format. For this reason, please be aware that any references listed in an SI file will not be indexed. For more information, see our Supporting Information guidelines:

https://journals.plos.org/plosbiology/s/supporting-information

*Published Peer Review History*

Please note that you may have the opportunity to make the peer review history publicly available. The record will include editor decision letters (with reviews) and your responses to reviewer comments. If eligible, we will contact you to opt in or out. Please see here for more details:

https://plos.org/published-peer-review-history/

*Press*

Should you, your institution's press office or the journal office choose to press release your paper, please ensure you have opted out of Early Article Posting on the submission form. We ask that you notify us as soon as possible if you or your institution is planning to press release the article.

*Protocols deposition*

To enhance the reproducibility of your results, we recommend that if applicable you deposit your laboratory protocols in protocols.io, where a protocol can be assigned its own identifier (DOI) such that it can be cited independently in the future. Additionally, PLOS ONE offers an option for publishing peer-reviewed Lab Protocol articles, which describe protocols hosted on protocols.io. Read more information on sharing protocols at https://plos.org/protocols?utm_medium=editorial-email&utm_source=authorletters&utm_campaign=protocols

Please do not hesitate to contact me should you have any questions.

Sincerely,

Roli

Roland Roberts, PhD

Senior Editor

rroberts@plos.org

PLOS Biology

------------------------------------------------------------------------

DATA POLICY:

You may be aware of the PLOS Data Policy, which requires that all data be made available without restriction: http://journals.plos.org/plosbiology/s/data-availability. For more information, please also see this editorial: http://dx.doi.org/10.1371/journal.pbio.1001797

Note that we do not require all raw data. Rather, we ask that all individual quantitative observations that underlie the data summarized in the figures and results of your paper be made available in one of the following forms:

1) Supplementary files (e.g., excel). Please ensure that all data files are uploaded as 'Supporting Information' and are invariably referred to (in the manuscript, figure legends, and the Description field when uploading your files) using the following format verbatim: S1 Data, S2 Data, etc. Multiple panels of a single or even several figures can be included as multiple sheets in one excel file that is saved using exactly the following convention: S1_Data.xlsx (using an underscore).

2) Deposition in a publicly available repository. Please also provide the accession code or a reviewer link so that we may view your data before publication.

Regardless of the method selected, please ensure that you provide the individual numerical values that underlie the summary data displayed in the following figure panels as they are essential for readers to assess your analysis and to reproduce it: Figs 1E, 2B, 3AB, 4BC, 5AB, S1, S2CDE, S3, S4AB, S5AB, S6ABC, S7ABC,S8ABC, S9ABC. NOTE: the numerical data provided should include all replicates AND the way in which the plotted mean and errors were derived (it should not present only the mean/average values).

IMPORTANT: Please also ensure that figure legends in your manuscript include information on where the underlying data can be found, and ensure your supplemental data file/s has a legend.

Please ensure that your Data Statement in the submission system accurately describes where your data can be found.

------------------------------------------------------------------------

CODE POLICY

Per journal policy, if you have generated any custom code during the course of this investigation, please make it available without restrictions. Please ensure that the code is sufficiently well documented and reusable, and that your Data Statement in the Editorial Manager submission system accurately describes where your code can be found. More information on our Code Policy, what and how to share can be found here: https://journals.plos.org/plosbiology/s/code-availability

Please note that we cannot accept sole deposition of code in GitHub, as this could be changed after publication. However, you can archive this version of your publicly available GitHub code to Zenodo. Once you do this, it will generate a DOI number, which you will need to provide in the Data Accessibility Statement (you are welcome to also provide the GitHub access information). See the process for doing this here: https://docs.github.com/en/repositories/archiving-a-github-repository/referencing-and-citing-content

------------------------------------------------------------------------

BLOT AND GEL REPORTING REQUIREMENTS:

We require the original, uncropped and minimally adjusted images supporting all blot and gel results reported in an article's figures or Supporting Information files. We will require these files before a manuscript can be accepted so please prepare and upload them now. Please carefully read our guidelines for how to prepare and upload this data: https://journals.plos.org/plosbiology/s/figures#loc-blot-and-gel-reporting-requirements

------------------------------------------------------------------------

DATA NOT SHOWN?

- Please note that per journal policy, we do not allow the mention of "data not shown", "personal communication", "manuscript in preparation" or other references to data that is not publicly available or contained within this manuscript. Please either remove mention of these data or provide figures presenting the results and the data underlying the figure(s).

------------------------------------------------------------------------

REVIEWERS' COMMENTS:

Reviewer #1:

I appreciate the response to the comments I have raised. I believe the paper should now be accepted as is.

Reviewer #2:

The authors have addressed all the concerns I raised. All minor issues have been corrected, and I therefore recommend acceptance of the manuscript.

# Editor report: Decision Letter 3

**DOI:** 10.1371/journal.pbio.3004065.r005

**Authors:** Roland Roberts

7 Oct 2026

Dear Susana,

Thank you for the submission of your revised Research Article "Life-cycle plasticity enables conditional asexual reproduction in a kelp" for publication in PLOS Biology. On behalf of my colleagues and the Academic Editor, Joe Heitman, I'm pleased to say that we can in principle accept your manuscript for publication, provided you address any remaining formatting and reporting issues. These will be detailed in an email you should receive within 2-3 business days from our colleagues in the journal operations team; no action is required from you until then. Please note that we will not be able to formally accept your manuscript and schedule it for publication until you have completed any requested changes.

Please take a minute to log into Editorial Manager at http://www.editorialmanager.com/pbiology/, click the "Update My Information" link at the top of the page, and update your user information to ensure an efficient production process.

PRESS: We frequently collaborate with press offices. If your institution or institutions have a press office, please notify them about your upcoming paper at this point, to enable them to help maximise its impact. If the press office is planning to promote your findings, we would be grateful if they could coordinate with biologypress@plos.org. If you have previously opted in to the early version process, we ask that you notify us immediately of any press plans so that we may opt out on your behalf.

We also ask that you take this opportunity to read our Embargo Policy regarding the discussion, promotion and media coverage of work that is yet to be published by PLOS. As your manuscript is not yet published, it is bound by the conditions of our Embargo Policy. Please be aware that this policy is in place both to ensure that any press coverage of your article is fully substantiated and to provide a direct link between such coverage and the published work. For full details of our Embargo Policy, please visit http://www.plos.org/about/media-inquiries/embargo-policy/.

Thank you again for choosing PLOS Biology for publication and supporting Open Access publishing. We look forward to publishing your study.

Sincerely,

Roli

Roland G Roberts, PhD, PhD

Senior Editor

PLOS Biology

rroberts@plos.org
